# Ligand-directed covalent labelling of a GPCR with a fluorescent tag in live cells

**DOI:** 10.1038/s42003-020-01451-w

**Published:** 2020-11-27

**Authors:** Leigh A. Stoddart, Nicholas D. Kindon, Omolade Otun, Clare R. Harwood, Foteini Patera, Dmitry B. Veprintsev, Jeanette Woolard, Stephen J. Briddon, Hester A. Franks, Stephen J. Hill, Barrie Kellam

**Affiliations:** 1 Cell Signalling and Pharmacology Research Group, Division of Physiology, Pharmacology and Neuroscience, School of Life Sciences, University of Nottingham, Nottingham, NG7 2RD UK; 2 Centre of Membrane Proteins and Receptors (COMPARE), University of Birmingham and University of Nottingham, Nottingham, Midlands NG7 2RD UK; 3 School of Pharmacy, Biodiscovery Institute, University of Nottingham, Nottingham, NG7 2RD UK; 4 Division of Cancer and Stem Cells, School of Medicine, University of Nottingham, Nottingham, NG7 2RD UK

**Keywords:** Cellular imaging, Receptor pharmacology

## Abstract

To study the localisation of G protein-coupled receptors (GPCR) in their native cellular environment requires their visualisation through fluorescent labelling. To overcome the requirement for genetic modification of the receptor or the limitations of dissociable fluorescent ligands, here we describe rational design of a compound that covalently and selectively labels a GPCR in living cells with a fluorescent moiety. We designed a fluorescent antagonist, in which the linker incorporated between pharmacophore (ZM241385) and fluorophore (sulfo-cyanine5) is able to facilitate covalent linking of the fluorophore to the adenosine A_2A_ receptor. We pharmacologically and biochemically demonstrate irreversible fluorescent labelling without impeding access to the orthosteric binding site and demonstrate its use in endogenously expressing systems. This offers a non-invasive and selective approach to study function and localisation of native GPCRs.

## Abstract (web summary)

Stoddart et al. propose rational design of a covalent and selective ligand probe to fluorescently label GPCR in transiently transfected and endogenous systems. Using the adenosine A_2A_ receptor as a model system, they show fluorescent labelling in an endogenous system, without impeding access to the orthosteric binding site. This study is useful to selectively and non-invasively study localisation and functions of GPCRs.

## Introduction

Fluorescent labelling a protein of interest can be achieved in many different ways and is an absolute prerequisite for many techniques used to probe both their function and localisation. For GPCRs, this is often achieved through use of protein tags (e.g., GFP, SNAP-tag), fluorescently labelled antibodies or fluorescent ligands^1^. Each of these technologies has its  limitations and they are not readily transferrable to endogenously expressing systems where GPCRs are expressed at low levels. Gene editing techniques, such as CRISPR/Cas9^2^, have allowed labelling of proteins^3^, including GPCRs^4,5^, at endogenous levels but the fluorophore used can dramatically alter expression levels^6^. An orthogonal approach to protein labelling uses ligand-directed chemistry where a fluorophore is connected via a highly reactive, electrophilic linker to a ligand that binds to the protein of interest. Upon binding of this conjugate, the linker can undergo a substitution reaction with a nucleophilic amino acid side chain (Lys, Ser, Thr, Tyr, Cys) close to the binding site, forming a new covalent bond between the fluorophore and protein^7^. The reactive groups used thus far for labelling proteins are bulky and not readily accommodated within the binding sites of a GPCR or suffer from slow reactivity^8,9^.

Here, using the adenosine A_2A_ receptor (A_2A_R) as a model system we describe the rational design of a compound (**1**) that can covalently fluorescently label the receptor in a ligand directed manner. Using a combination of pharmacological and biochemical approaches we demonstrate the covalent labelling of the A_2A_R with **1** and that labelling does not impede access to the orthosteric binding site. In addition, we demonstrate its use in labelling the A_2A_R in endogenously expressing systems.

## Results and discussion

### Rational design of ligand-directed probe for the A_2A_R

**Fig. 1:**
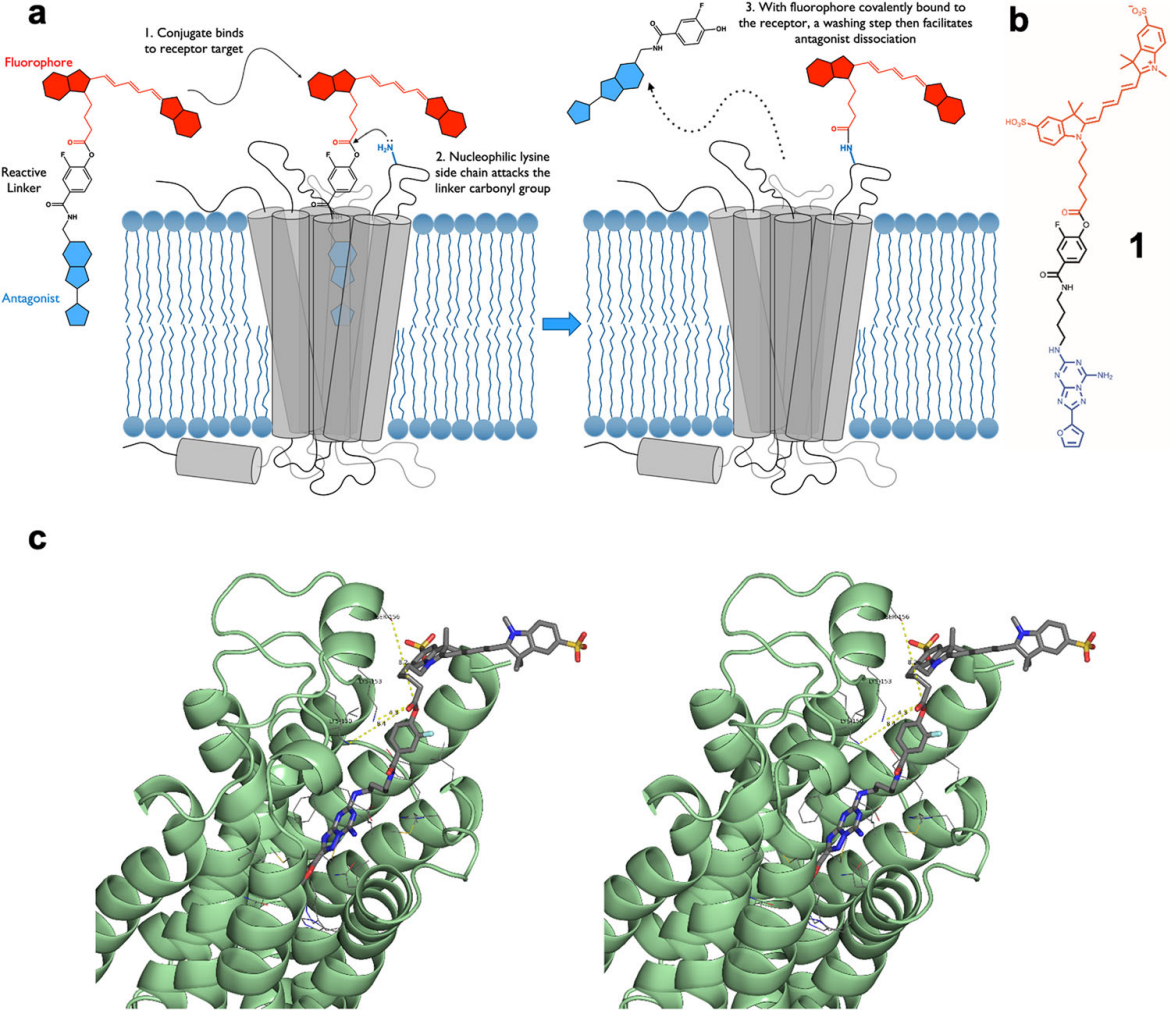
Schematic and molecular modelling of a ligand designed to covalently label A_2A_R. **a** Schematic illustration of ligand-directed labelling of A_2A_R by 1. **b** Chemical structure of **1**. **c** Cross-eyed stereo view of 1 docked into a refined model of the human A2a receptor (PDB: 5K2B) highlighting Lys153 as the closest nucleophilic amino acid residue (~4.3 Å).

To overcome the reactive group size and reactivity difficulties described for ligand-directed labelling approaches^8,9^, we incorporated a smaller phenyl ester group as the reactive moiety between the orthosteric head-group and fluorophore of a fluorescent conjugate for the A_2A_R, with the phenyl ring forming part of the binding pharmacophore (Fig. 1a). To enhance the chemical reactivity of the ester, molecular modelling suggested that the relatively small fluorine atom, the most electronegative element, could be introduced onto this phenyl ring and was compatible with binding of the antagonist into the binding site. If required, a further increase in chemical reactivity was envisaged by incorporating more fluorine atoms onto the phenyl ring. Based on recent fluorescent antagonists^10^ and the selective A_2A_R antagonist ZM241385^11^ we designed such a ligand, **1** (Fig. 1b, Supplementary Fig. 1), and used molecular modelling to guide close proximity of the phenyl ester with a nucleophilic amino acid side chain to allow transfer of a Cy5 fluorophore to the receptor (Fig. 1c, Supplementary Fig. 2). The majority of the ligand-receptor poses revealed a close proximity (4–7 Å) of the ε-amino group of Lys153 and the electrophilic region of the linker and that the fluorophore was in a predominately solvated state. Within the surrounding vestibule of the orthosteric binding pocket of the A_2A_R there are surprisingly few nucleophilic side chain residues and the approximate molecular distances between the electrophilic centre of the phenyl ester and the two nearest nucleophilic residues; Lys150 and Ser156 were found to be consistently 8–10 Å from the carbonyl of the phenyl ester.

### Pharmacological and biochemical characterisation of 1 in model cell systems

**Fig. 2:**
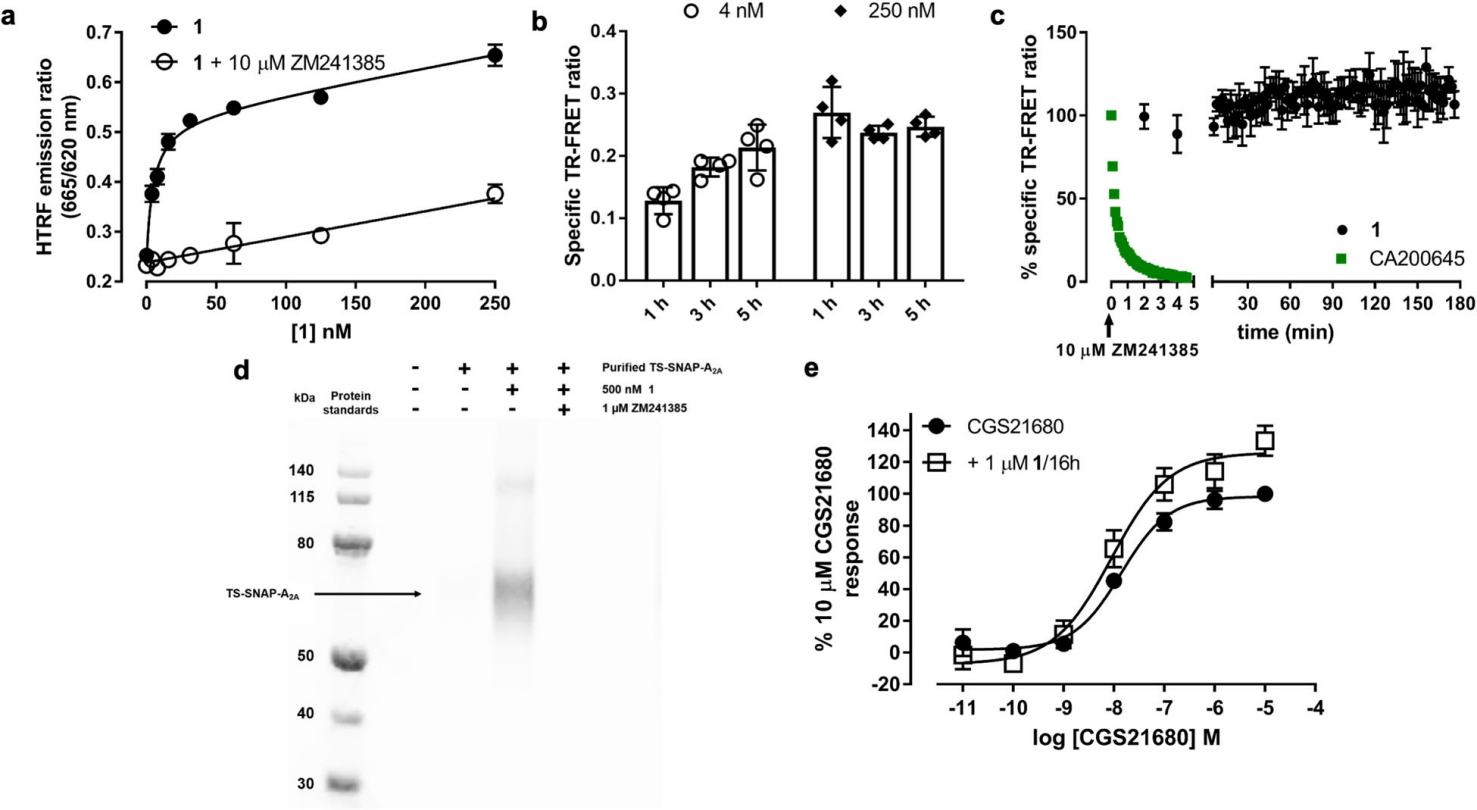
Pharmacological and biochemical characterisation of 1. **a** TR-FRET saturation binding curves obtained by treating membranes containing Lumi4-Tb labelled SNAP-A_2A_R with increasing concentrations of **1** in the absence (closed circles) or presence (open circles) of 10 µM ZM241385 for 1 h at 37 °C prior to determination of TR-FRET ratio. Data shown are representative of four experiments and each data point represents mean ± s.e.m of triplicate determinations. **b** TR-FRET measurements as detailed in **a** taken after 1, 3 and 5 h for 4 nM (open circles) and 250 nM (diamonds) **1**. Data shown  are specific TR-FRET ratios and represents mean ± SEM of four experiments performed in triplicate. **c** Membranes containing Lumi4-Tb labelled SNAP-A_2A_R were pre-treated with 250 nM **1** (5 h, black circles) or CA200645 (2 h, green squares) at 37 °C prior to measurement of TR-FRET ratio. After basal reads, 10 µM ZM241385 was added and measurements taken every 2 min (**1**) or 5 s (CA200645) for 5 min (CA200645) or 180 min (**1**). Non-specific binding was determined in the presence of 10 µM ZM241385 and data normalised to total and non-specific binding at the zero time point. Each data point represents mean ± s.e.m. of four experiments each performed in triplicate. **d** T-Rex^TM^-293 cells induced to express TS-SNAP-A_2A_R were treated with 500 nM **1** in the presence or absence of 1 µM ZM241385. Untreated cells were used as a control. TS-SNAP-A_2A_R was purified and separated on an SDS-PAGE gel and direct Cy5 fluorescence was visualised using in-gel fluorescence. Gel shown is representative of three independent experiments. **e** CHO CRE-SPAP cells were treated with (open squares) or without (closed circles) 1 µM **1** for 16 h. Cells were washed for 30 min prior to the addition of increasing concentrations of CGS21680 and levels of CRE-mediated SPAP production measured after 5 h. Data are normalised to basal (in the absence of agonist and **1**) and maximal CGS21680 response in the absence of **1** and each point represents the mean ± s.e.m. of five experiments performed in triplicate.

To test its labelling efficacy in an over-expression system, we added increasing concentrations of **1** to membranes prepared from HEK293 cells expressing SNAP-A_2A_R labelled with Lumi4-Tb and measured time-resolved fluorescence resonance energy transfer (TR-FRET) between Lumi4-Tb and the Cy5 subsequently attached to the receptor by **1** via ligand-directed covalent labelling. We detected a concentration-dependent increase in TR-FRET ratio which could be inhibited by co-incubation with the unlabelled competitive antagonist ZM241385 (10 µM; Fig. 2a). This was consistent with specific binding to the A_2A_R and resulted in an estimated equilibrium dissociation constant (K_D_) of 5.5 ± 2.0 nM for **1** (*n* = 4). As these measurements were taken after 1 h, to investigate if this was long enough to reach maximal labelling, TR-FRET measurements were also taken at 3 h and 5 h. Figure 2b demonstrates that there was no increase in the TR-FRET signal after 3 h and 5 h with 250 nM **1**, whereas with lower concentrations a time-dependent increase was seen. This is expected, as the association rate of the ligand (*k*_on_) is dependent on the concentration of ligand used, so for lower concentrations of ligand it will take longer to bind to all available receptors and subsequently label them. From these data, in further experiments the minimum labelling conditions were for 2 h with 250 nM **1**.

To test the irreversible nature of this binding interaction, we added an excess of ZM241385 (10 μM) to membranes previously labelled with 250 nM **1** and observed very little change in the TR-FRET ratio over the subsequent 3 h (103.2 ± 6.8% vs. 0 time point, *n* = 4). This suggested that the fluorophore remained in close proximity to the Lumi4-Tb labelled receptor and could not be displaced by ZM241385, indicating either permanent transfer of the flurophore or very slow dissociation of the ligand from the receptor (Fig. 2c). In marked contrast, when we undertook a similar experimental protocol with the competitive orthosteric fluorescent antagonist CA200645 (Supplementary Fig. 3a), the TR-FRET ratio returned to baseline within 5 min of addition of ZM241385 (Fig. 2c, Supplementary Fig. 3b) indicating full dissociation from the receptor. To confirm covalent labelling of A_2A_R by **1**, T-Rex^TM^-293 cells expressing a Twin-Strep-SNAP-A_2A_R (TS-SNAP-A_2A_R) construct were treated with **1** prior to purification, separation by SDS-PAGE and visualisation of labelled samples by in-gel fluorescence. A strong band corresponding to Cy5 labelled TS-SNAP-A_2A_R was observed at 73 kDa, the expected molecular weight of a monomer, and a concomitant weaker band at 130 kDa (Fig. 2d, Supplementary Fig. 4). Labelling of A_2A_R with Cy5 was prevented when the cells were co-incubated with **1** and ZM241385 (1 μM). The presence of Cy5 fluorescence after purification and denaturation of A_2A_R confirms the hypothesis that a covalent bond is present between receptor and fluorophore.

**1** was designed to label the A_2A_R with Cy5 and then allow dissociation of the pharmacophore portion of the ligand from the orthosteric binding site, enabling additional ligands to subsequently bind to the receptor. However, it is conceivable that labelling the receptor with Cy5 close to the entrance of the binding pocket may act as a structural barrier and prevent access of additional ligands^12^. The A_2A_R is a G_s_ coupled receptor and its activation leads to an increase in cAMP and subsequent transcription of genes under the control of a cAMP response element (CRE). Therefore, to determine whether the orthosteric binding site was accessible after Cy5 labelling of the receptor, we evaluated the ability of the A_2A_-selective agonist CGS21680 to stimulate CRE-mediated gene expression in CHO CRE-SPAP (secreted placental alkaline phosphatase) cells expressing the A_2A_R. In naïve cells, increasing concentrations of CGS21680 produced a concentration-dependent increase in SPAP production (pEC_50_ = 7.86 ± 0.09, *n* = 5). When cells were treated overnight (16 h) with 1 µM **1**, conditions which lead to a significant fluorescent labelling of the A_2A_R, and washed prior to addition of the agonist, CGS21680 remained equally potent compared to untreated cells (Fig. 2e, pEC_50_ = 8.05 ± 0.16, *n* = 5; *p* = 0.32, unpaired *t*-test). A small increase in the maximum response (13.3 ± 5.2% increase in maximal SPAP production vs. control, Fig. 2e) is unlikely to be biologically significant. These data demonstrate that following covalent labelling of the receptor by **1** the antagonist pharmacophore readily dissociates from the orthosteric receptor binding site. Furthermore, agonist access to the orthosteric binding site was not impaired by the covalent attachment of Cy5 to the receptor.

### Visualisation of labelling of A_2A_R by 1 in model cells

**Fig. 3:**
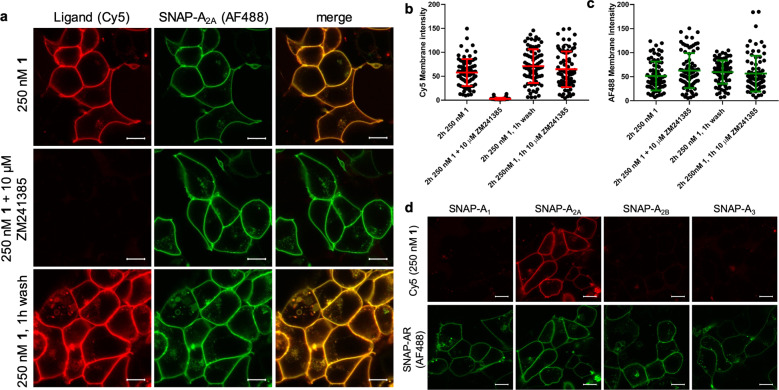
1 is selective and can be used to visualise A_2A_R in live cells. **a** Live HEK293 cells expressing SNAP-A_2A_R were labelled with SNAP-surface-AF488 and then treated with 250 nM **1** in the absence or presence of ZM241385 for 2 h prior to the capture of single equatorial confocal images. Cells treated with 250 nM **1** were then washed multiple times over 1 h and then imaged. Left hand column represents Cy5 fluorescence, middle column AF488 fluorescence and right hand column the merged image. Fluorescent intensity values (8-bit greyscale) were determined for Cy5 (**b**) and AF488 (**c**) in membrane regions of interest from the images obtained as described in **a** and also in cells treated with 10 μM ZM241385 for 1 h after 2 h incubation with 250 nM **1** (images shown in Supplementary Fig. 4). Each point represents the values obtained from one cell. Images were obtained in four independent experiments, error bars represent mean ± SD. **d** HEK293 cells expressing SNAP tagged versions of one of the four adenosine receptor subtypes (A_1_, A_2A_, A_2B_ or A_3_) were labelled with SNAP-surface-AF488 prior to the addition of 250 nM **1** for 2 h prior to the capture of single equatorial images. Images shown in **a** and **d** are representative of images taken in four (**a**) or three (**d**) independent experiments, with all image sets taken using identical settings for laser power, gain, and offset in both channels. Scale bar shown represents 10 µm.

We visualised the Cy5 labelling of SNAP-A_2A_R by **1** in live HEK293 cells using confocal microscopy. We observed clear membrane localisation of the Cy5 fluorescence in the continued presence of **1** and a high degree of co-localisation with the AF488 labelled SNAP-A_2A_R (Fig. 3a). This fluorescence labelling was prevented in the presence of ZM241385 (10 μM), demonstrating again that the ligand needs to bind to the orthosteric binding site of the A_2A_R in order to transfer the fluorescent label. After labelling with 250 nM **1** for 2 h, we observed no change in fluorescence intensity at the cell surface despite washing cells four times over 1 h (Fig. 3a–c). The addition of a high concentration of ZM241385 (10 μM) after pre-labelling with **1** also had no influence on the level of Cy5 fluorescence (Fig. 3b, Supplementary Fig. 5). These data demonstrate that **1** can label A_2A_R irreversibly with Cy5 in a live-cell system.

One of the advantages of ligand-directed labelling of proteins over other labelling methodologies is that selectivity between closely related proteins can be achieved through the use of a ligand that binds selectively to the protein of interest. To check the selectivity of **1** itself, we treated HEK293 cells expressing the three other adenosine receptor subtypes (SNAP-labelled A_1_R, A_2B_R, A_3_R) with **1** and determined the levels of labelling with Cy5 using confocal imaging (Fig. 3d). We observed no specific Cy5 fluorescence in cells expressing A_1_R, A_2B_R or A_3_R. This high degree of selectivity of **1** for A_2A_R over the other three adenosine receptor subtypes cannot be attributed solely to the selectivity of ZM241385 as it is reported to be only 60 fold selective for A_2A_R over A_2B_R^11^. It is therefore likely that this selectivity also results from close proximity of nucleophilic residues only present within A_2A_R to the reactive core of **1** when it is resident within the orthosteric binding site of the receptor (Supplementary Fig. 6).

### Ligand-directed labelling of endogenously expressed A_2A_R

**Fig. 4:**
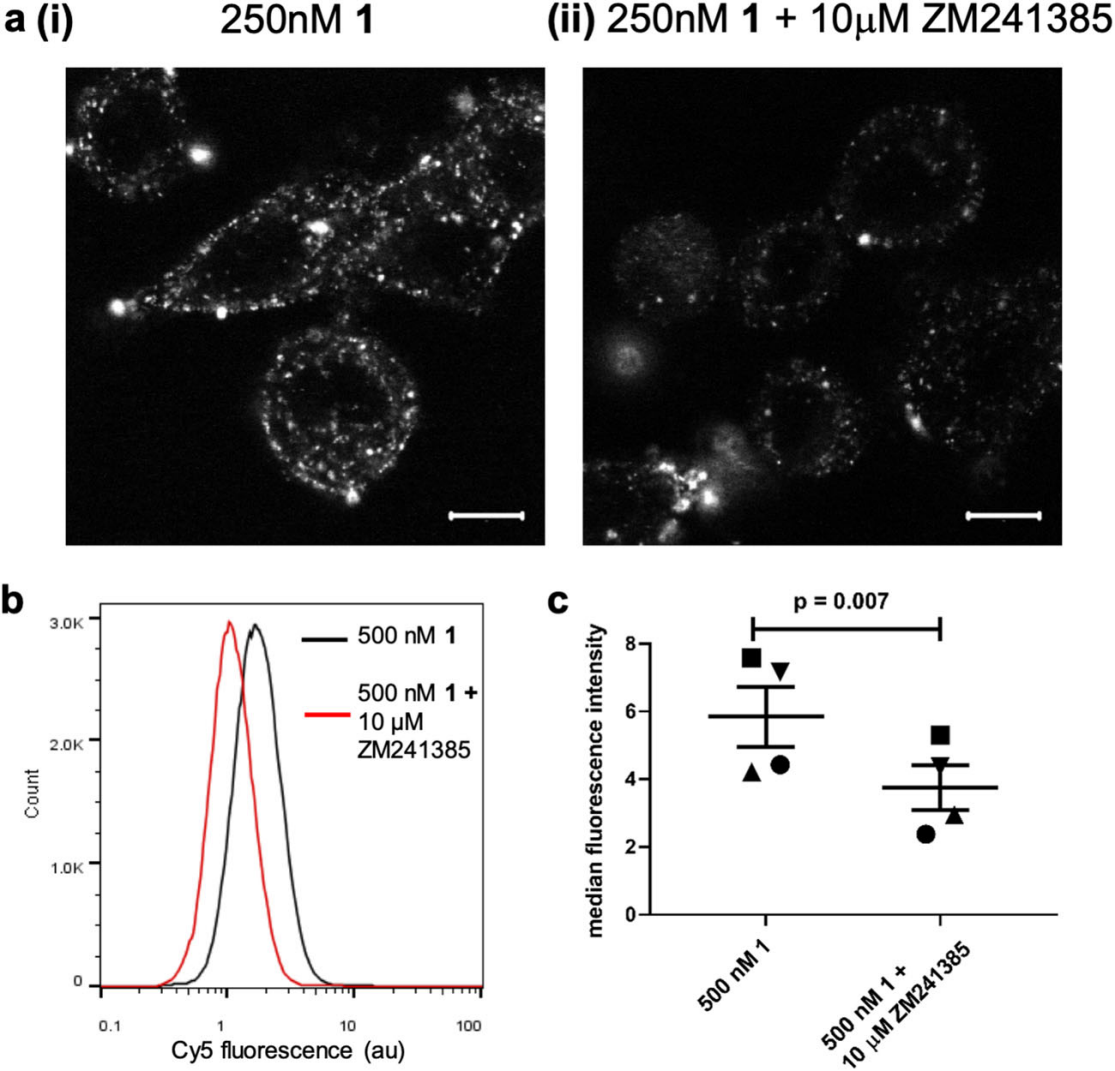
Labelling of endogenously expressed A_2A_R by 1. **a** SK-BR-3 cells were labelled with 250 nM **1** in the (ii) presence or (i) absence of 10 µM ZM241385 for 2 h prior to the capture of single equatorial confocal images. Images shown are representative of images taken in three independent experiments, with all image sets taken using identical settings for laser power, gain, and offset in both channels. Scale bar shown represents 10 µm. **b** Representative flow cytometry histograph of human macrophages treated with 500 nM **1** (black line) or 500 nM **1** plus 10 μM ZM241385 (red line) for 2 h at 37 °C. **c** Median fluorescence intensity of macrophages derived from four healthy donors treated as in **b** (*p* = 0.007, paired *t*-test). Each symbol represents one donor and line shows mean with s.e.m.

As **1** showed selectivity for A_2A_R over other adenosine receptor subtypes, we then investigated if it could be used to label endogenously expressed A_2A_Rs. For this we selected the human breast cancer cell line SK-BR-3 and human monocyte-derived macrophages which are both known to express the A_2A_R^13–15^. Live-cell confocal imaging of SK-BR-3 cells treated with **1** showed Cy5 labelling, which was prevented by the presence of ZM241385 (Fig. 4a). When compared to the imaging observed in the HEK293 cells overexpressing SNAP-A_2A_R (Fig. 3a), the labelling in the SK-BR-3 cells is more punctate. The SK-BR-3 cells have a flat morphology when compared HEK293 cells and as thus it is difficult to obtain a halo of plasma membrane labelling. In addition, there appears to be more intracellular clusters of receptors which may be due to labelled A_2A_R which has been constitutively trafficked by the vesicular machinery in the cells. In addition, ZM241385-senstitive labelling of macrophages with Cy5 was observed via flow cytometry (Fig. 4b, *p* < 0.0001, two-sided unpaired *T*-test with Welch’s correction; Fig. 4c, *p* = 0.007, two-sided paired *t*-test; Supplementary Fig. 7) demonstrating labelling of endogenous A_2A_R with **1**.

Ligand directed labelling of a GPCR compliments the very successful traditional labelling approaches such as the SNAP-tag^16^ and NanoLuc^17^ technologies. As an extension of studying GPCRs with fluorescent ligands, this means that each GPCR target requires a separate ligand-directed label to be developed^18^. With the explosion in the number of high-resolution structures solved^19^, the rational design of ligand-directed labels for GPCRs should be achievable through the use of molecular modelling to reduce the size of the reactive groups and gain selectivity over closely related receptor subtypes through careful positioning of these groups close to subtype-specific nucleophilic residues. An often-observed pitfall with fluorescent ligands is high levels of non-specific binding, which can occlude the detection of specific binding in endogenously expressing systems^20^. As multiple wash steps can be performed in a ligand-directed labelling approach, this has the potential to reduce the levels of non-specific binding observed. Fluorescent ligands^21–23^ and the recently described ligand-directed label for the μ opioid receptor^24^ have been used to visualise the organisation and expression pattern of endogenously expressed receptors. Due to the proposed role of the A_2A_R in neurological conditions such as Parkinson’s disease^25^ and as a target for cancer immunotherapy^26^, the approach described here has the potential to allow the function of the A_2A_R in normal and disease conditions to be studied.

In summary, we have described rational design of a compound that can covalently and selectively label a GPCR with a florescent molecule without affecting the binding site of the receptor. Ligand directed labelling of GPCRs is a non-invasive approach to visualise GPCRs and opens up the possibilities to study ligand binding, receptor trafficking and signalling in endogenously and clinically relevant systems.

## Methods

Chemicals and solvents of analytical and HPLC grade were purchased from commercial suppliers and used without further purification. Sulfo-Cyanine5 carboxylic acid was purchased from Lumiprobe. Reactions were monitored by thin-layer chromatography on commercially available silica pre-coated aluminium-backed plates (Merck Kieselgel 60 F254). Visualisation was under UV light (254 nm and 366 nm). Flash column chromatography was performed using silica gel 60, 230–400 mesh particle size (Sigma Aldrich). NMR spectra were recorded on a Bruker-AV 400. ^1^H NMR spectra were recorded at 400.13 MHz and ^13^C NMR spectra at 126.00 MHz. Chemical shifts (δ) are recorded in parts per million (ppm) and coupling constants are recorded in Hz. The following abbreviations are used to described signal shapes and multiplicities; singlet (s), doublet (d), triplet (t), quadruplet (q), broad (br), dd (doublet of doublets), ddd (double doublet of doublets), dtd (double triplet of doublets) and multiplet (m). Processing of the NMR data was carried out using the NMR software Topspin. LC-MS spectra were recorded on a Shimadzu UFLCXR system coupled to an Applied Biosystems API2000 and visualised at 254 nm (channel 1) and 220 nm (channel 2). LC-MS was carried out using a Phenomenex Gemini-NX C18 110 A, column (50 mm × 2 mm × 3 μm) at a flow rate 0.5 mL/min over a 5 min period. All high resolution mass spectra (HRMS) were recorded on a Bruker microTOF mass spectrometer using MS electrospray ionisation operating in positive or negative ion mode. RP-HPLC was performed on a Waters 515 LC system and monitored using a Waters 996 photodiode array detector at wavelengths between 190 and 800 nm. Spectra were analysed using Millenium 32 software. Semi-preparative HPLC was performed using YMC-Pack C8 column (150 mm × 10 mm × 5 μm) at a flow rate of 5.0 mL/min using a gradient method of 20–70% B over 15 min (Solvent A = 0.1% formic acid in H_2_O, solvent B = 0.1% formic acid in CH_3_CN). Analytical RP-HPLC was performed using a YMC-Pack C8 column (150 mm × 4.6 mm × 5 μm) at a flow rate of 1.0 mL/min. Final products were one single peak and >98% pure. The retention time of the final product is reported using a gradient method of 10–95% solvent B in solvent A over 26 min. (Solvent A = 0.1% formic acid in H_2_O, solvent B = 0.1% formic acid in CH_3_CN).

### 2-(Furan-2-carbonyl)hydrazine-1-carboximidamide (3)

2-Furoic hydrazide (**2**) (6.0 g, 47.5 mmol) and 2-methyl-2-thiopseudourea hemisulfate salt (33.1 g, 238 mmol) were added to a stirred solution of sodium hydroxide (3.04 g, 76 mmol) in water (150 ml) at room temperature. After 24 h, the precipitate was collected by filtration and washed with water and diethyl ether to give 2-(furan-2-carbonyl)hydrazine-1-carboximidamide^27^. Yield 4.00 g (23.8 mmol, 50%) and used directly in the next step without further purification.

### 3-(Furan-2-yl)-1*H*-1,2,4-triazol-5-amine (4)

2-(Furan-2-carbonyl)hydrazine-1-carboximidamide (**3**) (4.0 g, 23.8 mmol) was suspended in water (70 mL) and the mixture heated under reflux for 24 h^27^. The reddish solution was evaporated to dryness and the resulting solid slurried in water, collected by filtration and dried to give 3-(furan-2-yl)-1*H*-1,2,4-triazol-5-amine^27^. Yield 2.8 g (18.6 mmol, 78%). ^1^H NMR (400 MHz, DMSO) δ 12.07 (s, 1H), 7.68 (s, 1H), 6.68 (d, *J* = 3.3 Hz, 1H), 6.54 (s, 1H), 6.07 (s, 2H).

### 2-(Furan-2-yl)-5-(methylthio)-[1,2,4]triazolo[1,5-*a*][1,3,5]triazin-7-amine (5)

Dimethyl *N*-cyanodithioiminocarbonate (2.2 g, 15 mmol) and 3-(furan-2-yl)-1*H*-1,2,4-triazol-5-amine (2.2 g, 14.6 mmol) were mixed and heated at 170 °C under nitrogen for 1 h^27^. After cooling the crude reaction mixture was dissolved in methanol and dichloromethane and absorbed on to silica. Purification was by silica gel chromatography eluting with 5–10% ethyl acetate in dichloromethane to give 2-(furan-2-yl)-5-(methylthio)-[1,2,4]triazolo[1,5-*a*][1,3,5]triazin-7-amine^27^. Yield 1.4 g (5.6 mmol, 38%). LC/MS (*m*/*z*) 248.9 (M + 1), r.t. 2.47 min, ^1^H NMR (400 MHz, DMSO) δ 9.11–8.54 (br s, 2H), 7.93 (dd, *J* = 1.8, 0.8 Hz, 1H), 7.17 (dd, *J* = 3.4, 0.8 Hz, 1H), 6.72 (dd, *J* = 3.4, 1.8 Hz, 1H), 2.52 (s, 3H).

### 2-(Furan-2-yl)-5-(methylsulfonyl)-[1,2,4]triazolo[1,5-*a*][1,3,5]triazin-7-amine (6)

3-chloroperbenzoic acid (3.8 g, 22 mmol) was added portionwise over 3 min to a stirred suspension of 2-(furan-2-yl)-5-(methylthio)-[1,2,4]triazolo[1,5-*a*][1,3,5]triazin-7-amine (1 g, 4.0 mmol) in dichloromethane (100 mL)^27^. Initially a clear solution formed and then a precipitate. After 16 h, the mixture was concentrated to 10 ml and ethanol (10 mL) added. The mixture was concentrated to 10 mL and the product allowed to crystallise out over 3 h which was collected by filtration to give 2-(furan-2-yl)-5-(methylsulfonyl)-[1,2,4]triazolo[1,5-*a*][1,3,5]triazin-7-amine^27^. Yield 0.9 g (3.2 mmol, 80%). LC/MS (*m*/*z*) 281.2 (M + 1), r.t. 2.12 min, ^1^H NMR (400 MHz, DMSO) δ 9.81 (s, 1H), 9.48 (s, 1H), 7.99 (dd, *J* = 1.8, 0.8 Hz, 1H), 7.27 (dd, *J* = 3.4, 0.8 Hz, 1H), 6.76 (dd, *J* = 3.5, 1.8 Hz, 1H), 3.37 (s, 3H).

### 2-(Furan-2-yl)-[1,2,4]triazolo[1,5-*a*][1,3,5]triazin-5-yl)amino)butyl)carbamate (7)

*tert*-Butyl *N*-(4-aminobutyl)carbamate (0.3 g, 1.6 mmol) in acetonitrile (1.5 ml) was added to a stirred solution of 2-(furan-2-yl)-5-(methylsulfonyl)-[1,2,4]triazolo[1,5-*a*][1,3,5]triazin-7-amine(0.3 g, 1.07 mmol) in acetonitrile (3 ml). After 2 h, the reaction mixture was absorbed onto isolute and applied to a silica gel column eluting with 5–10% methanol in dichlormethane to give tert-butyl (4-((7-amino-2-(furan-2-yl)-[1,2,4]triazolo[1,5-*a*][1,3,5]triazin-5-yl)amino)butyl)carbamate. Yield 0.3 g (0.78 mmol, 73%). ^1^H NMR (400 MHz, DMSO) δ 8.25-7.93 (m, 2H), 7.87 (d, *J* = 1.8 Hz, 1H), 7.54–7.35 (m, 1H), 7.04 (d, *J* = 3.6 Hz, 1H), 6.82–6.77 (m, 1H), 6.68 (dd, *J* = 3.5, 1.9 Hz, 1H), 3.30-3.19 (m, 2H), 2.93 (q, *J* = 6.5 Hz, 2H), 1.53–1.46 (m, 2H), 1.45–1.39 (m, 2H), 1.37 (s, 9H).

### *N*-(4-((7-amino-2-(furan-2-yl)-[1,2,4]triazolo[1,5-*a*][1,3,5]triazin-5-yl)amino)butyl)-3-fluoro-4-hydroxybenzamide (8)

*tert*-Butyl (4-((7-amino-2-(furan-2-yl)-[1,2,4]triazolo[1,5-*a*][1,3,5]triazin-5-yl)amino)butyl)carbamate (0.035 g, 0.09 mmol) in dichloromethane (5 ml) was treated with trifluoroacetic acid (2 ml). After 35 min the solution was evaporated to dryness. Toluene (10 ml) was added and the mixture evaporated to dryness. This process was repeated 2 more times to remove residual trifluoracetic acid to give *N*^5^-(4-aminobutyl)-2-(furan-2-yl)-[1,2,4]triazolo[1,5-*a*][1,3,5]triazine-5,7-diamine trifluoroacetate salt which was used without further purification.

1-[Bis(dimethylamino)methylene]-1*H*-1,2,3-triazolo[4,5-*b*]pyridinium 3-oxide hexafluorophosphate (HATU) (0.041 g, 0.1 mmol) was added to a stirred solution of 3-fluoro-4-hydroxybenzoic acid (0.014 g, 0.09 mmol) and diisopropylethylamine (0.15 ml) in DMF (1 ml). After 0.5 h, the slurry was added to the *N*^5^-(4-aminobutyl)-2-(furan-2-yl)-[1,2,4]triazolo[1,5-*a*][1,3,5]triazine-5,7-diamine trifluoroacetate salt and diisopropylethylamine (0.25 ml) in DMF (1 ml). The mixture was heated at 90 °C for 2 h. After cooling, the solvent was removed under high vacuum. Purification was by silica gel chromatography eluting with 5–10% methanol in dichloromethane to give *N*-(4-((7-amino-2-(furan-2-yl)-[1,2,4]triazolo[1,5-*a*][1,3,5]triazin-5-yl)amino)butyl)-3-fluoro-4-hydroxybenzamide. Yield 0.03 g (0.07 mmol, 70%) as a white solid. LC/MS (*m/z*) 427.6 (M + 1), r.t. 2.31 min, ^1^H NMR (400 MHz, DMSO) δ 10.42 (s, 1H), 8.30 (t, *J* = 5.6 Hz, 1H), 8.20-8.00 (br s, 2H), 7.87 (d, *J* = 1.8 Hz, 1H), 7.63 (dd, *J* = 12.3, 2.1 Hz, 1H), 7.55 (dd, *J* = 8.4, 2.1 Hz, 1H), 7.55-7.40 (m, 1H), 7.04 (d, *J* = 3.5 Hz, 1H), 6.98 (t, *J* = 8.6 Hz, 1H), 6.68 (dd, *J* = 3.5, 1.8 Hz, 1H), 3.32-3.20 (m, 4H), 1.59-1.53 (m, 4H). ^13^C NMR (126 MHz, DMSO) δ 165.19, 161.65, 159.68, 156.27, 150.81 (d, J 240 Hz) 150.44, 148.09 (d, J 11.4 Hz), 146.70, 145.08, 126.39 (d, J 5.4 Hz), 124.60 (d, J 2.52 Hz), 117.55 (d, 3.78 Hz), 115.57 (d, J 18.9 Hz), 112.37, 112.05, 27.20, 26.88. (2 × CH_2_ under DMSO peak)

### 2-((1*E*,3*E*)-5-((*E*)-1-(6-(4-((4-((7-Amino-2-(furan-2-yl)-[1,2,4]triazolo[1,5-*a*][1,3,5]triazin-5-yl)amino)butyl)carbamoyl)-2-fluorophenoxy)-6-oxohexyl)-3,3-dimethyl-5-sulfoindolin-2-ylidene)penta-1,3-dien-1-yl)-1,3,3-trimethyl-3*H*-indol-1-ium-5-sulfonate (1)

2-Bromo-1-ethyl-pyridinium tetrafluoroborate (BEP reagent)(0.32 mg, 1.17 × 10^−6^ mol) and diisopropylethylamine (10 mg, 7.75 × 10^−5^ mol) in dry *N*,*N*-dimethylformamide (0.6 ml) was added to sulfo-cyanine-C5 carboxylic acid (1 mg, 1.47 × 10^−6^ mol). After 15 min in the dark, this solution was added to *N*-(4-((7-amino-2-(furan-2-yl)-[1,2,4]triazolo[1,5-a][1,3,5]triazin-5-yl)amino)butyl)-3-fluoro-4-hydroxybenzamide (**8**) (0.7 mg, 1.6 × 10^−6^ mol). After 16 h in the dark, the solution was evaporated to dryness under high vacuum. Purification was by semi-preparative RP-HPLC. The product containing fractions were combined and concentrated to remove most of the acetonitrile then lyophilised to give 2-((1*E*,3*E*)-5-((*E*)-1-(6-(4-((4-((7-amino-2-(furan-2-yl)-[1,2,4]triazolo[1,5-*a*][1,3,5]triazin-5-yl)amino)butyl)carbamoyl)-2-fluorophenoxy)-6-oxohexyl)-3,3-dimethyl-5-sulfoindolin-2-ylidene)penta-1,3-dien-1-yl)-1,3,3-trimethyl-3*H*-indol-1-ium-5-sulfonate as a blue solid. Yield 1.0 mg (9.5 × 10-7 mol, 65%). HMRS (m/z) M-H calculated for C_51_H_55_FN_10_O_10_S_2_: 1049.3455, found M-H: 1049.3452. Analytical RP-HPLC, retention time 13.80 min.

### Molecular docking general method

The refined three-dimensional model of the human A2a receptor (pdb code: 5K2B) was obtained from the GPCRdb (https://www.gpcrdb.org) and loaded into SeeSAR docking software (SeeSAR version 10.1; BioSolveIT GmbH, Sankt Augustin, Germany, 2020, www.biosolveit.de/SeeSAR). Prior to docking, the binding site was established using the bound ligand ZM241385 and waters were excluded. Compound **1** was drawn in ChemDraw Professional 19.1 (Perkin Elmer Informatics Inc.) and imported into SeeSAR via a.mol file. FlexX docking of **1** was undertaken using ZM241385 as a template, with the 10 best scoring poses returned using the HYDE scoring function and visually inspected. Outputs were exported and visualised using PyMOL (Version 1.8 Schrödinger, LLC).

### cDNA constructs

We generated SNAP-labelled adenosine receptor constructs by amplifying the full length sequence of SNAP-tag (New England Biolabs, Ipswich, MA) and fusing it in frame with the membrane signal sequence of the 5HT_3A_ receptor with pcDNA3.1 to yield sig.SNAP. We then fused the full-length human sequence of each of the four adenosine receptor subtypes (with the methionine start signal removed) to the 3’ end of the sig.SNAP in pcDNA3.1. This gave the constructs designated as SNAP-A_1_R, SNAP-A_2A_R, SNAP-A_2B_R and SNAP-A_3_R, all of which contain the signal sequence. We generated the pcDNA4TO-TS-SNAP-A_2A_ construct by amplifying the A_2A_ receptor (with the methionine start signal removed) and inserting into a pcDNA4TO vector (ThermFisher Scientific, Paisley, UK) containing a Twin-Step-tag^®^ sequence and the SNAP sequence using Gibson assembly^28^. This gave the construct designated as TS-SNAP-A_2A_R. Plasmids will be made available to other researchers on request to the corresponding authors.

### Ligands

CA200645 was from HelloBio (Bristol, UK). ZM 241385 and CGS21680 were from Tocris (Bristol, UK).

### Cell culture and transient transfection

Human embryonic kidney 293 (HEK293) cells expressing the GloSensor cAMP biosensor (HEKG) were obtained from Promega (Southampton, UK) and were maintained in Dulbecco’s modified Eagle’s medium (DMEM) containing 10% foetal calf serum (FCS) and L-glutamine. T-REx^TM^-293 cells (Invitrogen) stably expressing the pcDNA4TO TwinStrepSNAP-A_2A_ (TS-SNAP-A_2A_) construct were maintained in high glucose DMEM containing 10% FCS, 5 μg/μL blasticidin and 20 μg/μL zeocin. Chinese hamster ovary (CHO) cells stably expressing a cAMP response element-secreted placental alkaline phosphatase (CRE-SPAP) reporter were maintained in Dulbecco’s Modified Eagle Medium:Nutrient Mixture F-12 (DMEM/F12) medium containing 10% FCS and 2 mM L-glutamine. SK-BR-3 cells obtained from ATCC (Manassas, VA) were maintained in McCoy’s 5a Medium supplemented with 10% FCS. All cell lines were maintained at 37 °C in a humidified atmosphere of air/CO_2_ (19:1). For transient transfections, HEK293G cells grown in 8-well chamber slides were transfected with the required SNAP-adenosine receptor construct using Fugene (Promega) according to the manufacturers’ instructions 24 h prior to experiment. For SNAP-A_2A_AR stable cell line, HEK293G cells were transfected with the SNAP-A_2A_AR construct using Fugene and cells expressing the construct were selected by the addition of 1 mg/mL^−1^ G418 to the normal growth media for 2–3 weeks. For A_2A_AR CRE-SPAP stable cell line, CHO CRE-SPAP cells were transfect with un-tagged A_2A_AR (Missouri S&T cDNA Resource Centre, MO, USA) using Lipofectamine (Life Technologies, Paisley, UK) according to the manufacturers’ instructions. Cells expressing A_2A_AR were selected by the addition of 1 mg/ml^−1^ G418 to normal growth media for 2–3 week and then dilution-cloned to obtain cell lines originating from a single cell.

### Human macrophage generation

Heparinised whole blood was obtained by venepuncture from the antecubital fossa of the arm of healthy volunteers after written informed consent (Ethics from University of Nottingham Ethics committee, ref 161–1711). Peripheral blood mononuclear cells (PBMC) were immediately separated by density centrifugation over Ficoll Histopaque 1077 (Sigma) followed by washes in endotoxin-free phosphate-buffered saline (PBS, Sigma). PBMC were washed in MACS buffer (PBS + 1% foetal calf serum (FCS, Sigma) + 2 µM EDTA (Sigma)) then incubated with CD14 microbeads (Miltenyi Biotech) and monocytes isolated by magnetic separation (purity routinely >95%). Purified CD14 + monocytes were differentiated into macrophages at 37 °C/5%CO_2_ for 7 days at 1 × 10^6^/well in low-attachment 24-well plates (Corning Costar) in macrophage medium (RPMI 1640 (Sigma) supplemented with 10% FCS and 1% sodium pyruvate (Sigma)) and 20U/mL recombinant human granulocyte-macrophage colony-stimulating factor (Peprotech) with medium plus additives supplemented at day 4.

### Macrophage 1 labelling and flow cytometry

Medium was aspirated from day 7 macrophages and 500 nM **1** in the presence or absence of 10 µM ZM241385 added in fresh macrophage medium whilst cells remained in the differentiation plate. Cells were incubated for 2 h at 37 °C/5% CO_2_ then medium aspirated, wells washed with cold PBS and macrophages harvested by incubating on ice in cold PBS for 25 min. Cy5 fluorescence was measured by flow cytometry (MacsQuant, Miltenyi Biotech) and analysed using FlowJo software. Gating strategy is demonstrated in Supplementary Fig. 7.

### Lumi4-Tb labelling and membrane preparation

SNAP-A_2A_ HEKG cells were grown to confluence in poly-D-lysine coated T175 flasks. Cells were washed once in PBS, 100 nM SNAP-Lumi4-Tb in 1× Lab Med (CisBio, Bagnols-sur-Ce’ze, France) was added and incubated for 1 h at 37 °C. After 1 h, SNAP-Lumi4-Tb solution was removed and replaced with ice-cold PBS, and the cells were removed from the flask using a cell scraper. The cells were then transferred to a 50 mL tube and centrifuged at 250 × *g* for 10 min. The supernatant was discarded and the resulting pellets were stored at −80 °C. For membrane preparation, cell pellets were thawed and resuspended in ice-cold PBS and homogenised using an IKA T10 Ultra-Turrax disperser in 10 × 5 s bursts at 15,000 rpm. After removal of unbroken cells and nuclei by centrifugation at 1200 × *g* for 10 min, the resulting supernatant was centrifuged at 41,415 × *g* for 30 min to obtain the membrane pellet. The pellet was then resuspended in ice-cold PBS and homogenised by 20 passes at 1000 rpm using a Kartell serrated pestle and a borsilicate glass homogeniser mortar fitted to an IKA RW16 overhead stirrer. Protein concentration of the resuspended membranes was determined using a bicinchoninic acid protein assay and SNAP-Lumi4-Tb membranes stored at −80 °C until required.

### TR-FRET binding assay

All TR-FRET assays were performed in opaque bottomed 96-well plates and read on a PHERAstar FS (BMG Labtech, Offenberg, Germany) with the terbium (donor) excited with 30 flashes of laser at 337 nm and emission collected at 620 nm (terbium) and 665 nm (Cy5/BY630) 400 ms after excitation. The TR-FRET ratio was calculated by dividing the Cy5/BY630 emission (665 nM) by the terbium emission (620 nm). For membrane saturation TR-FRET binding assays, 2.5 μg of Lumi4-Tb labelled SNAP-A_2A_ membranes were incubated with the required compounds in HEPES buffered saline solution (HBSS: 145 mmol/L NaCl, 5 mmol/L KCl, 1.7 mmol/L CaCl_2_, 1 mmol/L MgSO_4_, 10 mmol/L HEPES, 2 mmol/L sodium pyruvate, 1.5 mmol/L NaHCO_3_, 10 mmol/L D-glucose, pH 7.4) containing 1 mg/ml saponin for 1 h at 37 °C before reading on the PHERAstar. For dissociation experiments, 2.5 μg of SNAP-A_2A_R membranes were incubated with compounds in HBSS plus saponin for 5 h for **1** and 2 h for CA200645 at 37 °C in 96-well plates. After required incubation time, basal TR-FRET readings were taken on the PHERAstar, for **1**-treated membranes 10 μM ZM241385 was added to each well manually in a 1:1 dilution to ensure adequate mixing and TR-FRET readings were then taken every 5 min for 3 h as detailed above. For CA200645, 10 µM ZM241385 was added using the inbuilt PHERAstar injectors. Due to the rapid dissociation of CA200645, readings were taken every 5 s for 5 min with 20 flashes per read.

### Labelling of cells with 1 for purification and in gel fluorescence

TS-SNAP-A_2A_ TREx-293 cells were grown to 70% confluence prior to induction of TS-SNAP-A_2A_ expression by the addition of 1 μg/mL tetracyclin to normal growth medium. After 50 h induction, medium was replaced and to the required flasks 500 nM **1** or 500 nM **1** plus 1 µM ZM241385 added and cells incubated for a further 5 h at 37 °C/5% CO_2._ After 5 h, medium was removed and cells washed twice with phosphate buffered saline (PBS). Cells were then detached from flasks using cell dissociation solution non-enzymatic (Sigma), washed off with PBS and solutions removed from the flasks. Cell suspensions were spun at 362 × *g* for 10 min. Supernatant was aspirated and cell pellets frozen at −80 °C until use.

### Solubilisation and purification of 1 labelled TS-SNAP-A_2A_

Cell pellets were thawed on ice, weighed and resuspended in solubilisation buffer (1% n-Dodecyl β-D-maltoside (DDM) (w/v), 20 mM HEPES, 10% (v/v) glycerol, 150 mM NaCl, pH 7.5) at a ratio of 1:10 (w/v) of cell pellet to solubilisation buffer. Pellets were solubilised for 1 h on a DigiRoll 6 roller (SLS, UK) at 80RPM and 4 °C. Samples were clarified by centrifugation at 4122 × *g* for 20 min at 4 °C. Purification of TS-SNAP-A_2A_ was achieved by the use of MagStrep “type3” XT magnetic beads (IBA, Göttingen, Germany). Beads were prepared by removal of supernatant using a magnetic separator (IBA, Göttingen, Germany) and then they were washed twice in solubilisation buffer before being added to samples. Samples were incubated with beads overnight on a DigiRoll 6 roller set to 80 RPM at 4 °C. The following morning supernatant was removed from beads using the magnetic separator and beads were washed twice with wash buffer (0.1% DDM (w/v), 10% glycerol (v/v), 150 mM NaCl, pH 7.5), before resuspension in 30 μL elution buffer (1:9 solution of 10x buffer BXT (IBA) and wash buffer). Elution took place for 5 h on a DigiRoll 6 roller set to 80 RPM at 4 °C. Samples were then separated from beads using magnetic separator and then immediately processed for electrophoresis.

### Gel electrophoresis and in gel fluorescence

Fifteen microlitre of samples containing purified TS-SNAP-A_2A_ were mixed with 5 μL NuPAGE^TM^ LDS sample buffer and resolved on a NuPage^TM^ 4–12% Bis-Tris 15 × 1.0 mm well gel using NuPage^TM^ MOPs SDS running buffer. Gels were run for 50 min at 200 V. Samples were not boiled prior to gel electrophoresis. 5 μL PageRuler^TM^ Prestained Protein Ladder was used as the ladder. Gels were scanned on an Amersham Typhoon imaging system (GE Healthcare Life Sciences, Pittsburgh, PA) using Fluorstage and Cy5 670BP30 filter sets with PMT set to auto and pixel size to 200 μm. After fluorescence was measured, gels were stained with InstantBlue® protein stain (Expedeon, Cambridge, UK) overnight. Gels were washed twice with dH2O for 5 min before visualising using a standard smart phone camera.

### CRE-SPAP gene transcription

A_2A_R CRE-SPAP cells were grown to confluence in clear 96-well plates and on the day prior to analysis normal growth medium was removed and replaced with serum-free medium (SFM; DMEM/F12 supplemented with 2 mM L-glutamine) with or without 1 μM **1**. On the day of the experiment, all media was removed from wells and fresh SFM media added. Cells were incubated for 30 min at 37 °C, and then for the **1** overnight treated wells, SFM was removed and replaced with fresh SFM. Then increasing concentrations of CGS21680 was added to the required well and plates incubated for 5 h at 37 °C, 5% CO_2_. After the 5 h incubation, all medium was removed from the cells and replaced with 40 μl of SFM and incubated for a further 1 h at 37 °C. The plates were then incubated at 65 °C for 30 min to destroy the endgoneous alkaline phosphatases. After cooling the plates to room temperature, 5 mM 4-nitrophenyl phosphate in a diethanolamine-containing buffer (10% (v/v) diethanolamine, 280 mM NaCl, 500 μM MgCl_2_, pH 9.85) was added to each well. Plates were incubated for 15 min at 37 °C and then the absorbance at 405 nm was measured using a Dynex MRX plate reader (Chelmsford, MA, USA).

### Confocal microscopy

All confocal microscopy was performed on cells grown on 8-well Labtek chambered coverglasses (Nunc Nalgene). Where appropriate, cells were labelled with 100 nM SNAP-surface Alexa Fluor 488 (SNAP-AF488; New England Biolabs) for 30 min at 37 °C/5% CO_2_ in normal growth media. Cells were washed twice in media prior to the addition of the required compounds (250 nM **1** or 250 nM **1** + 10 μM ZM241385) in normal growth media and incubated for 2 h at 37 °C/5%CO_2_ and then imaged. Where required 10 μM ZM241385 was added after 2 h and cells incubated for a further 1 h prior to imaging. For wash experiments, cells were imaged after 2 h and then washed every 15 min for 1 h in normal growth media prior to imaging again. SK-BR3 cells were incubated with or without 10 μM ZM241385 in normal growth media for 30 min prior to the addition of 250 nM **1** and incubated for 2 h at 37 °C/5% prior to imaging. All imaging was performed using a Zeiss LSM880 confocal microscope (Carl Zeiss GmbH, Jena, Germany) using a ×63 plan-Apochromat NA 1.4 Ph3 oil-immersion objective and a 488/561/633 main dichoroic. For SNAP-AF488 a 488 nm argon laser was used to excite the fluorophore and the emission was detected between 493 and 628 nm. Cy5 was excited using a 633 nm HeNe laser and emission collected between 638 and 747 nm for HEKG cells and between 633 and 695 nm for SK-BR-3 cells. Due to the lower level of expression in the SK-BR3 cells, a pixel dwell time of 4.12 μs was used, whereas a pixel dwell time of 2.06 μs was used with HEKG cells. Within each experiment, a pinhole diameter of 1 Airy unit was used and the laser power, gain and offset kept constant for each experiment set. To obtain membrane intensity values, regions of interest were drawn by hand around the cell membranes in Zen Black image analysis software.

### Data analysis

We simultaneously fit the total and nonspecific saturation binding curves using Eq. 1:

1 $${\mathrm{TR}} - {\mathrm{FRET}}\;{\mathrm{ratio}} = \frac{{B_{{\mathrm{max}}}.\left[ B \right]}}{{\left[ B \right] + K_{\mathrm{D}}}} + \left( {\left( {M.[B]} \right) + C} \right)$$

where *B*_max_ is the maximal signal, [B] is the concentration of flurescent ligand in nM, *K*_D_ is the equilibrium dissociation constant in nM, *M* is the slope of the non-specific binding component and *C* is the intercept with the *y*-axis.

The CRE-SPAP data were fit to Eq. 2:

2 $${\mathrm{Response}} = \frac{{E_{{\mathrm{max}}}.\left[ A \right]}}{{\left[ A \right] + EC_{50}}}$$

where *E*_max_ is the maximal response, [*A*] is the concentration of agonist and the *EC*_50_ is the molar concentration of agonist required to generate 50% of the *E*_max_

### Statistics and reproducibility

The number of replicates for each experiment is given in the respective figure legend and was always greater than three. A replicate was defined as an assay performed on a separate day with a different passage of cells, transfection or donor.

We carried out statistical analysis using two-tailed paired or unpaired t test as required.

### Reporting summary

Further information on research design is available in the Nature Research Reporting Summary linked to this article.

## Supplementary information

Supplementary Information

Peer Review File

Description of Additional Supplementary Files

Supplementary Data 1

Supplementary Data 2

Supplementary Data 3

Reporting Summary

## Data Availability

The authors declare that all reported data in the main and supplementary files will be provided to other investigators as requested from the corresponding authors. Source data underlying all figures are provided in Supplementary Data 1. Raw image files, original gel images and source data for all figures can also be found on Mendeley Data^29^ (10.17632/8p56bzyg8z). The structure of **1** in Fig. 1b is provided in Supplementary Data 2. The reaction scheme in Supplementary Information is provided in Supplementary Data 3.
