# Supplementary material for: Ligand-directed covalent labelling of a GPCR with a fluorescent tag in live cells
Source: Commun Biol. 2020 Nov 27;3:722. doi: 10.1038/s42003-020-01451-w (PMC7695831; doi:10.1038/s42003-020-01451-w)
Supplement: Supplementary file 1 — Supplementary Information [file 42003_2020_1451_MOESM1_ESM.pdf]

Supplementary Information

Ligand-directed covalent labelling of a GPCR with a fluorescent tag in live cells

Leigh A Stoddart[1,2,5], Nicholas D Kindon[2,3,5], Omolade Otun[1,2], Clare R. Harwood[1,2], Foteini Patera[4], Dmitry B Veprintsev[1,2], Jeanette Woolard[1,2], Stephen J Briddon[1,2], Hester A Franks[2,4], Stephen J Hill[1,2*], Barrie Kellam[2,3*].

[1] Cell Signalling and Pharmacology Research Group, Division of Physiology, Pharmacology and Neuroscience, School of Life Sciences, University of Nottingham, Nottingham, UK.

[2] Centre of Membrane Proteins and Receptors (COMPARE), University of Birmingham and University of Nottingham, Midlands, UK.

[3] School of Pharmacy, Biodiscovery Institute, University of Nottingham, Nottingham, UK.

[4] Division of Cancer and Stem Cells, School of Medicine, University of Nottingham, Nottingham, UK

[5] These authors contributed equally to this work

* e-mail: stephen.hill@nottingham.ac.uk; barrie.kellam@nottingham.ac.uk

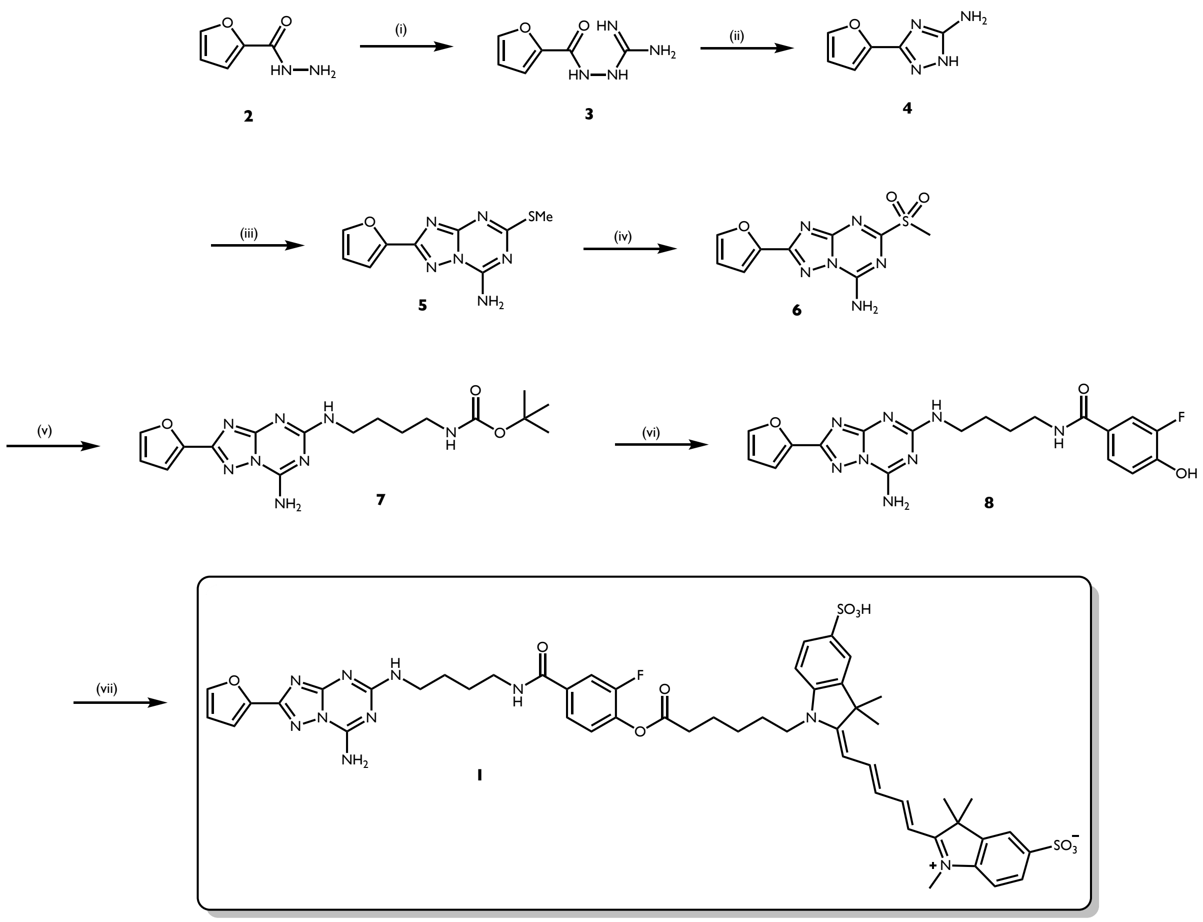

Supplementary Figure 1. Synthesis Scheme for Fluorescent Probe 1

Reagents and Conditions: (i) 2-methyl-2-thiopseudourea hemisulfate salt, $NaOH_{(aq)}$, 24 h; (ii) H_2O, **Δ**, 24 h; (iii) Dimethyl *N*-cyanodithioiminocarbonate, 170 ℃, 1 h; (iv) 3-chloroperbenzoic acid, dichloromethane, 16 h; (v) *tert*-Butyl *N*-(3-aminobutyl)-carbamate, acetonitrile, 2 h; (vi) (a) Trifluoracetic acid, dichloromethane, 0.5 h; (b) 3-fluoro-4-hydroxybenzoic acid, 1-[Bis(dimethylamino)methylene]-1*H*-1,2,3-triazolo[4,5-*b*]pyridinium 3-oxide hexafluorophosphate, diisopropylethylamine, *N,N*-dimethylformamide, 90 ℃, 2 h; (vii) sulfo-cyanine-C5 carboxylic acid, 2-Bromo-1-ethyl-pyridinium tetrafluoroborate, diisopropylethylamine, *N,N*-dimethylformamide, 16 h in the dark.

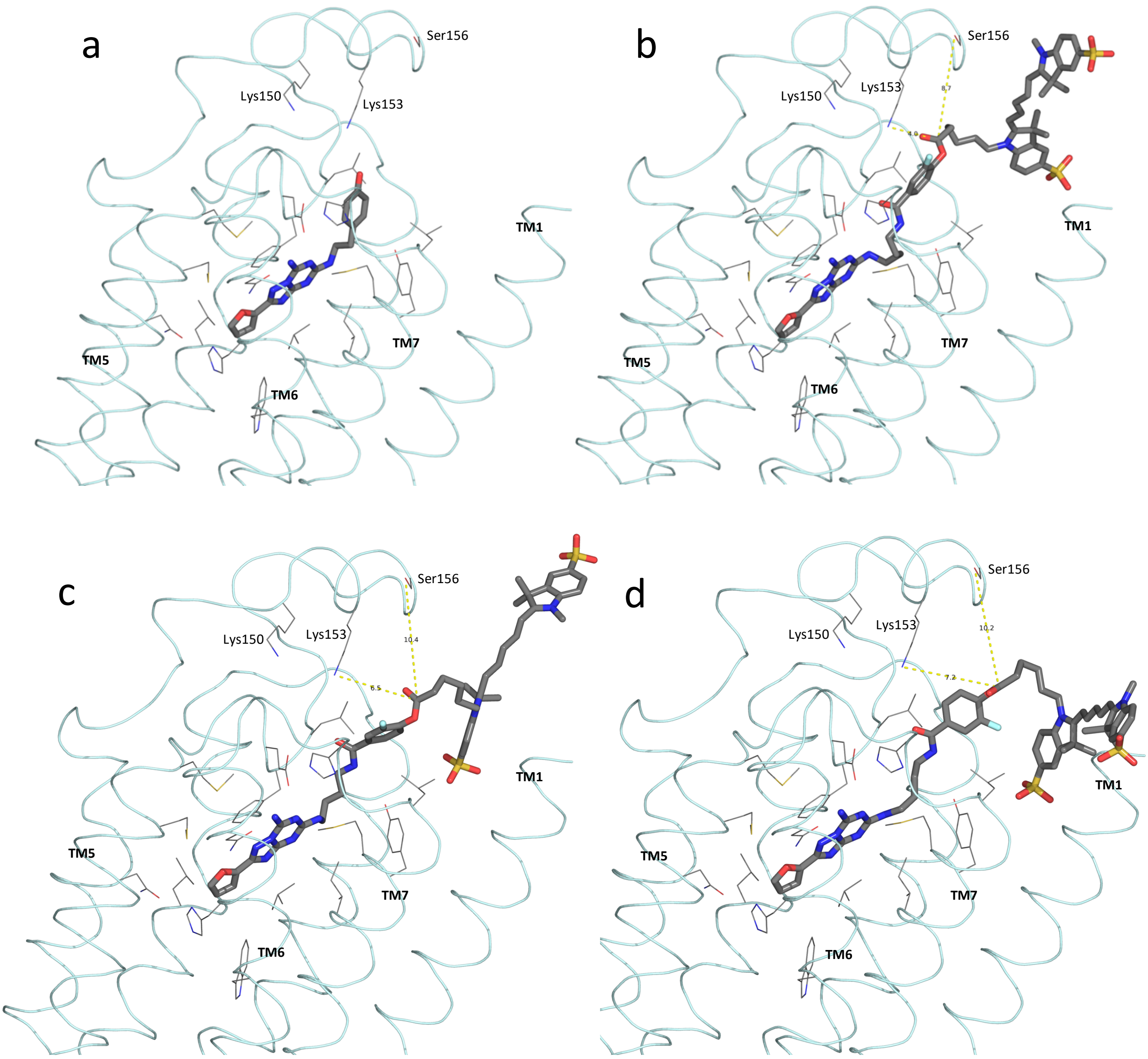

Supplementary Figure 2. Representation of the binding modes of ZM241385 and 1 in the human A_{2A} Receptor ($hA_{2a}R$). (a) ZM241385 bound in the $hA_{2A}R$ (green ribbon, Refined model of PDB: 5K2B; https://www.gpcrdb.org); **(b-d)** Three illustrative docking poses of **1** in the $hA_{2A}R$ (green ribbon, Refined model of PDB: 5K2B).

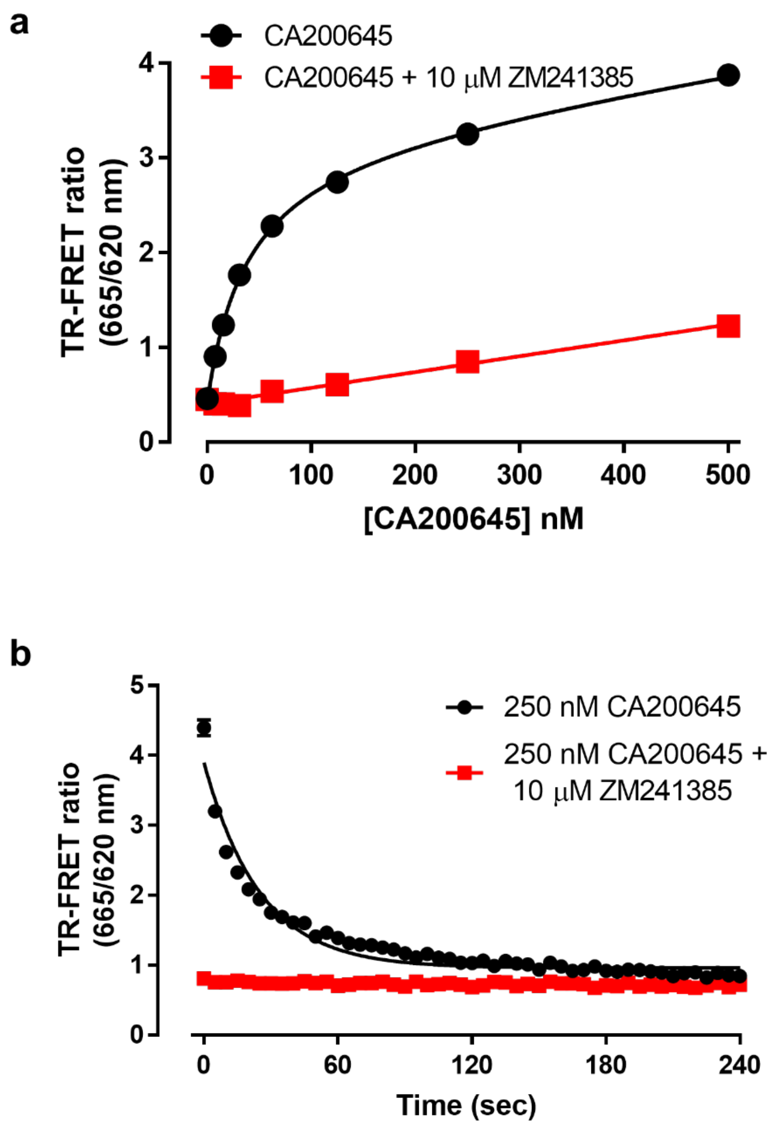

Supplementary Figure 3. Pharmacological characterisation of CA200645 at SNAP-A_{2A} (**a**) TR-FRET saturation binding curves obtained by treating membranes containing Lumi4-Tb labelled SNAP-A_{2A}R with increasing concentrations of CA200645 in the absence (black circles) or presence (red squares) of 10 μM ZM241385 for 1h at 37°C prior to determination of TR-FRET ratio. Data shown is representative of five experiments and each data point represents mean ± s.e.m of triplicate determinations. Calculated K_d for CA200645 at SNAP-A_{2A} was 36.8 ± 5.7 nM.(**b**) Membranes containing Lumi4-Tb labelled SNAP-A_{2A}R were pre-treated with 250 nM CA200645 in the presence (red squares) or absence (black circles) of 10 μM ZM241385 for 2h green squares at 37°C prior to measurement of TR-FRET ratio. After basal reads, 10 μM ZM241385 was added to all wells and measurements taken every 5 sec for 5 min. Data shown is representative of four experiments and each data point represents mean ± s.e.m of triplicate measurements.

(a)

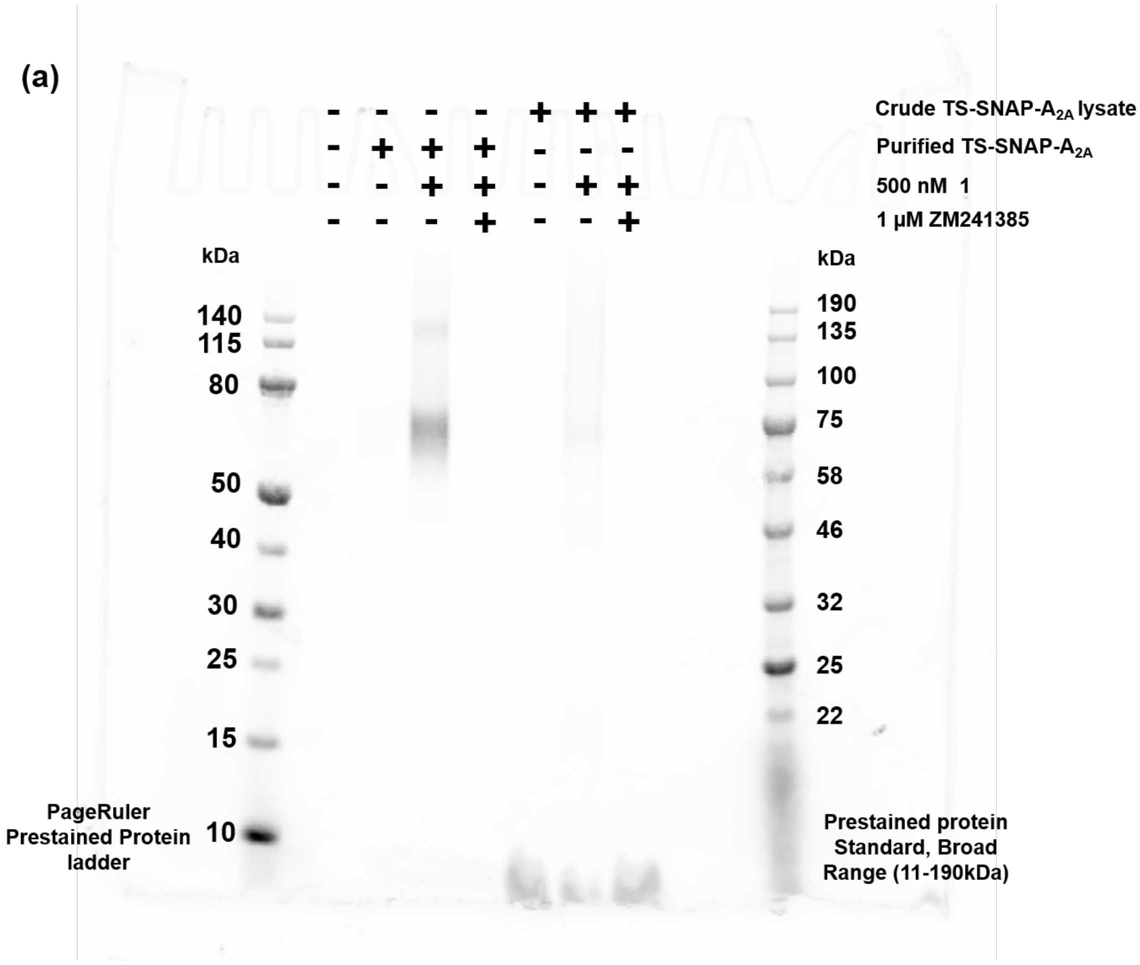
Crude TS-SNAP-A2A lysate
Purified TS-SNAP-A2A
500 nM 1
1 μM ZM241385
- - - - + + +
- + + + - - -
- - + + - + +
- - - + - - +
kDa
140
115
80
50
40
30
25
15
10
PageRuler Prestained Protein ladder
kDa
190
135
100
75
58
46
32
25
22
Prestained protein Standard, Broad Range (11-190kDa)

(b)

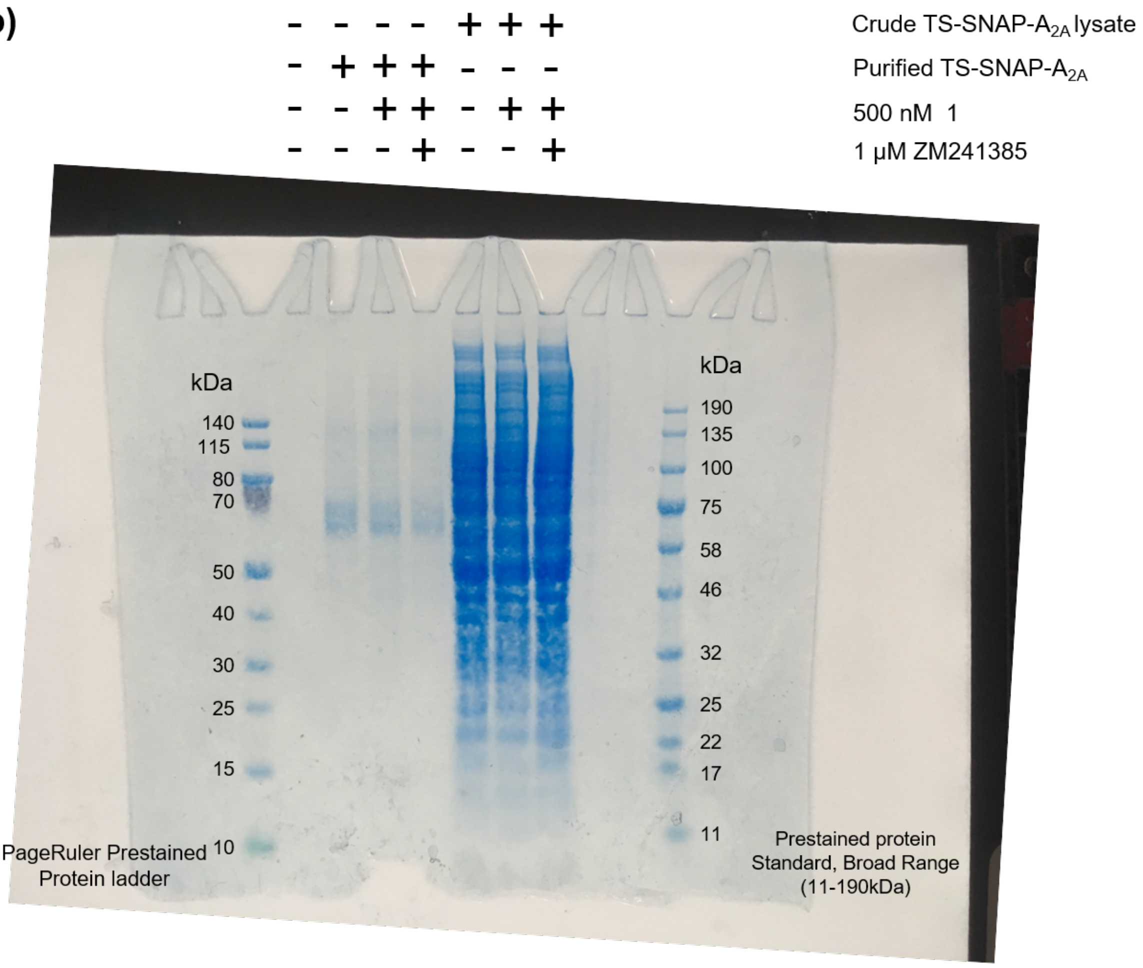

Supplementary Figure 4. Protein stain of 1 labelled samples

T-Rex™-293 cells induced to express TS-SNAP-A_{2A}R were treated with 500nM **1** in the presence or absence of 1µM ZM241385. Crude cell pellet extract from untreated cells before and after purification were used as controls. Where indicated TS-SNAP-A_{2A}R was purified and all samples were analysed on an SDS-PAGE gel. (a) Direct Cy5 fluorescence was visualised using in-gel fluorescence and cropped image shown in Figure 1e. After in-gel fluorescence was visualised, gel was stained for using InstantBlue® protein stain to demonstrate equal loading and purification and image obtained on a standard smartphone camera (b). Gel shown is representative of three independent experiments.

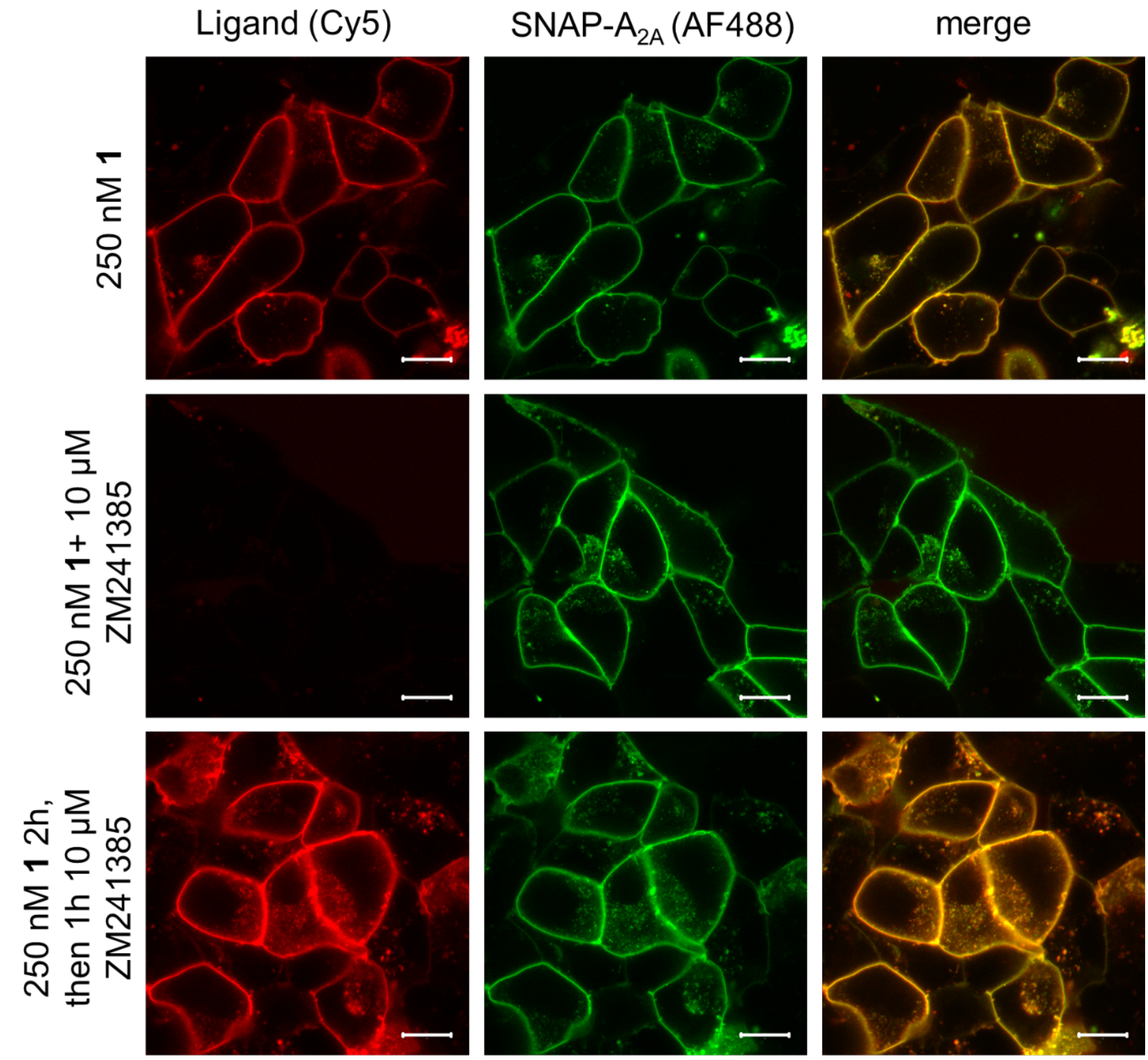

Supplementary Figure 5. Visualisation of Cy5 labelling with 1 after treatment with ZM241385 Live HEK293 cells expressing SNAP-A_{2A}R were labelled with SNAP-surface-AF488 and then treated with 250 nM **1** in the absence (top row) or presence (middle row) of ZM241385 for 2h prior to the capture of single equatorial confocal images. To the cells treated with 250 nM **1**, 10 µM ZM241385 was added and incubated for a further 1h then imaged (bottom row). Left hand column represents Cy5 fluorescence, middle column AF488 fluorescence and right hand column the merged image. Images shown are representative of images taken in four independent experiments, with all image taken using identical settings for laser power, gain, and offset in both channels. Scale bar shown equals 10 µm.

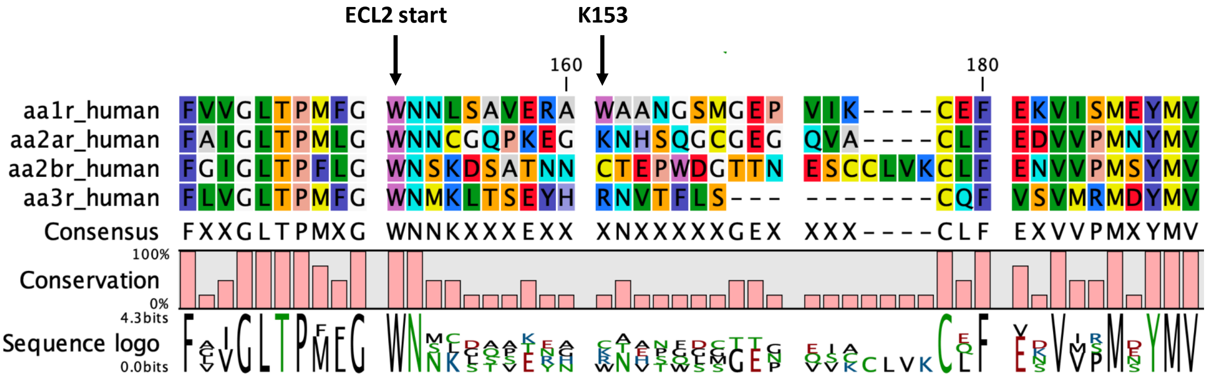

Supplementary Figure 6 Sequence alignment of extra cellular loop 2 of the four adenosine receptor subtypes

Alignment of the top of transmembrane region 5, extra-cellular loop 2 (ECL2), and top of transmembrane region 6 of the four adenosine receptor subtypes. The potential site of Cy5 attachment to K153, by **1** in the $A_{2A}R$ is indicated by an arrow which is not conserved in the other three adenosine receptor subtypes.

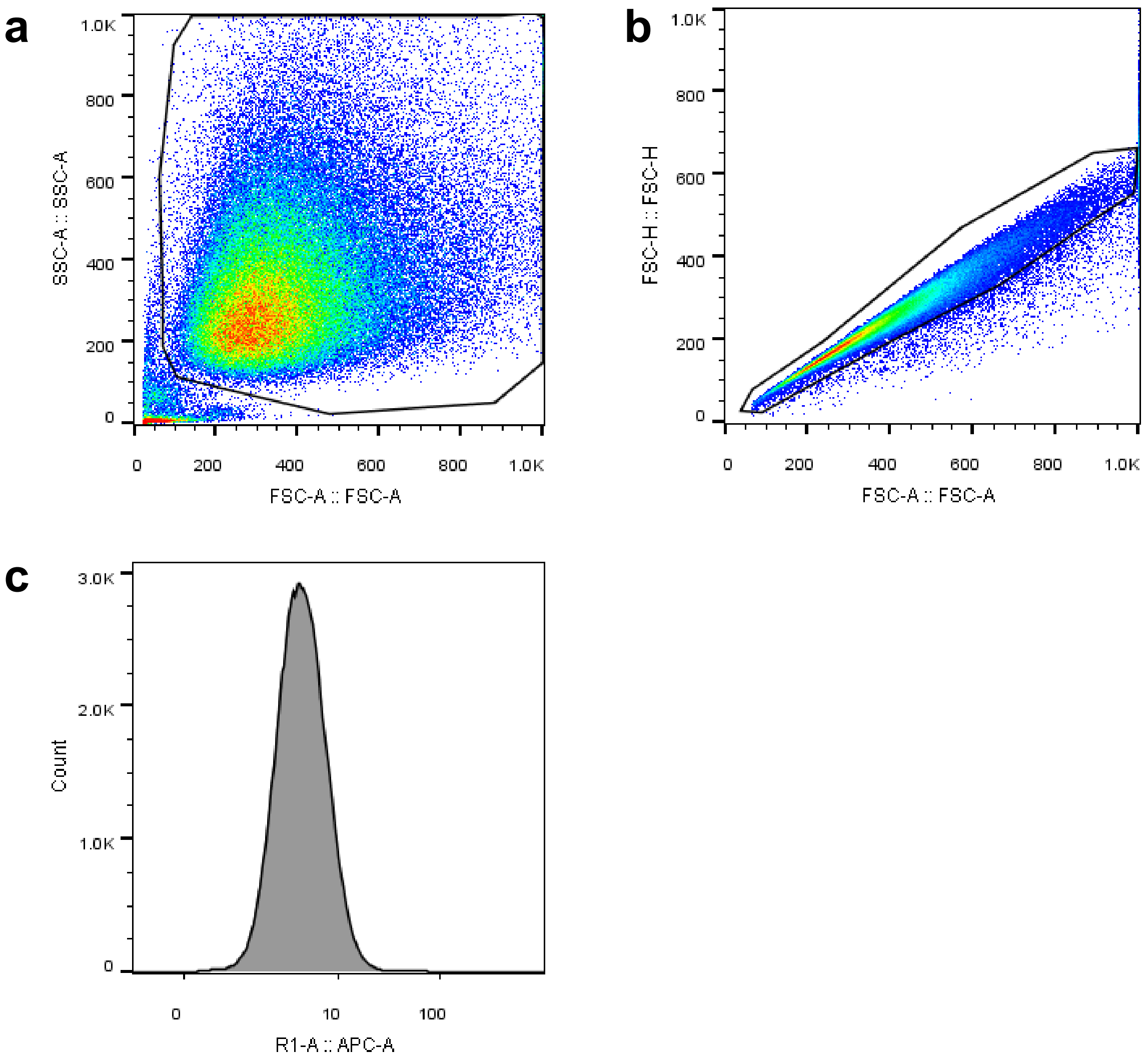

Supplementary figure 7 FACS gating strategy for macrophages

Macrophages were harvested on ice after incubating with **1** in the presence or absence of 10 μM ZM241385. AFSC/SSC plot was used to gate out debris (a), followed by Doublet exclusion (b) and singlet macrophages plotted as a histogram in R1 channel ('APC') for Cy5 fluorescence from NDK174 (c). Example shown is a representative sample (**1** + 10 μM ZM241385) from a representative donor (one donor of four).
