# Supplementary material for: Ligand-directed covalent labelling of a GPCR with a fluorescent tag in live cells
Source: Commun Biol. 2020 Nov 27;3:722. doi: 10.1038/s42003-020-01451-w (PMC7695831; doi:10.1038/s42003-020-01451-w)
Supplement: Supplementary file 3 — Description of Additional Supplementary Files [file 42003_2020_1451_MOESM3_ESM.pdf]

Description of Additional Supplementary Files

Title: Supplementary Data 1

Description: Excel spreadsheets for the data included in Figures 2, 3 and 4.

Title: Supplementary Data 2.

Description: ChemDraw molecule file (cdxml) for the structure of compound 1 shown in Figure 1b.

Title: Supplementary Data 3.

Description: ChemDraw file (cdx) for the reaction scheme shown in Supplementary Figure 1.
