# Supplementary material for: Ligand-directed covalent labelling of a GPCR with a fluorescent tag in live cells
Source: Commun Biol. 2020 Nov 27;3:722. doi: 10.1038/s42003-020-01451-w (PMC7695831; doi:10.1038/s42003-020-01451-w)
Supplement: Supplementary file 7 — Reporting Summary [file 42003_2020_1451_MOESM7_ESM.pdf]

nature research

Corresponding author(s): Barrie Kellam, Stephen J Hill

Last updated by author(s): Sep 26, 2020

Reporting Summary

Nature Research wishes to improve the reproducibility of the work that we publish. This form provides structure for consistency and transparency in reporting. For further information on Nature Research policies, see our Editorial Policies and the Editorial Policy Checklist.

Statistics

For all statistical analyses, confirm that the following items are present in the figure legend, table legend, main text, or Methods section.

n/a	Confirmed	
☐	☒	The exact sample size (*n*) for each experimental group/condition, given as a discrete number and unit of measurement
☐	☒	A statement on whether measurements were taken from distinct samples or whether the same sample was measured repeatedly
☐	☒	The statistical test(s) used AND whether they are one- or two-sided<br>*Only common tests should be described solely by name; describe more complex techniques in the Methods section.*
☒	☐	A description of all covariates tested
☐	☒	A description of any assumptions or corrections, such as tests of normality and adjustment for multiple comparisons
☐	☒	A full description of the statistical parameters including central tendency (e.g. means) or other basic estimates (e.g. regression coefficient) AND variation (e.g. standard deviation) or associated estimates of uncertainty (e.g. confidence intervals)
☐	☒	For null hypothesis testing, the test statistic (e.g. *F*, *t*, *r*) with confidence intervals, effect sizes, degrees of freedom and *P* value noted<br>*Give P values as exact values whenever suitable.*
☒	☐	For Bayesian analysis, information on the choice of priors and Markov chain Monte Carlo settings
☒	☐	For hierarchical and complex designs, identification of the appropriate level for tests and full reporting of outcomes
☒	☐	Estimates of effect sizes (e.g. Cohen's *d*, Pearson's *r*), indicating how they were calculated

Our web collection on statistics for biologists contains articles on many of the points above.

Software and code

Policy information about availability of computer code

Data collection	Zen Black; ChemDraw; Amersham Typhoon Scanner; BMG Pherastar Control Software and Mars. Miltenyi MACSquant.
Data analysis	GraphPad Prism 8.0; Zen Black; Flowjo

For manuscripts utilizing custom algorithms or software that are central to the research but not yet described in published literature, software must be made available to editors and reviewers. We strongly encourage code deposition in a community repository (e.g. GitHub). See the Nature Research guidelines for submitting code & software for further information.

Data

Policy information about availability of data

All manuscripts must include a data availability statement. This statement should provide the following information, where applicable:

- Accession codes, unique identifiers, or web links for publicly available datasets
- A list of figures that have associated raw data
- A description of any restrictions on data availability

The data that support the findings of this study are available on Mendeley Data and from the corresponding authors upon reasonable request.

Field-specific reporting

Please select the one below that is the best fit for your research. If you are not sure, read the appropriate sections before making your selection.

☒ Life sciences ☐ Behavioural & social sciences ☐ Ecological, evolutionary & environmental sciences

For a reference copy of the document with all sections, see nature.com/documents/nr-reporting-summary-flat.pdf

Life sciences study design

All studies must disclose on these points even when the disclosure is negative.

Sample size	We used power analysis on the basis of the standard deviation obtained in similar experiments to determine the probability of observing a small change in the parameter under study and determined that n greater than or equal to three was a sufficient sample size.
Data exclusions	None
Replication	Minimum of triplicates in n separate experiments.
Randomization	No formal randomization was undertaken due to the nature of the data being collected.
Blinding	Blinding was not practical in the experiments undertaken in this study.

Reporting for specific materials, systems and methods

We require information from authors about some types of materials, experimental systems and methods used in many studies. Here, indicate whether each material, system or method listed is relevant to your study. If you are not sure if a list item applies to your research, read the appropriate section before selecting a response.

Materials & experimental systems

n/a	Involved in the study	
☒	☐	Antibodies
☐	☒	Eukaryotic cell lines
☒	☐	Palaeontology and archaeology
☒	☐	Animals and other organisms
☐	☒	Human research participants
☒	☐	Clinical data
☒	☐	Dual use research of concern

Methods

n/a	Involved in the study	
☒	☐	ChIP-seq
☐	☒	Flow cytometry
☒	☐	MRI-based neuroimaging

Eukaryotic cell lines

Policy information about cell lines

Cell line source(s)	HEK293 expressing the GloSensor cAMP biosensor were obtained from Promega. T-REx-293 cells were obtained from Invitrogen and SK-BR3 cells were obtained from ATCC.
Authentication	None of the cell lines have been authenticated since they were obtained directly from commercial sources.
Mycoplasma contamination	HEK293-SNAP-A2A and HEK293-Glosensor cell lines were tested for mycoplasma contamination in August 2020 and were clear.
Commonly misidentified lines (See ICLAC register)	No commonly misidentified lines used in this study.

Human research participants

Policy information about studies involving human research participants

Population characteristics	Healthy donors.
Recruitment	Volunteers in response to group email requests for donors. Written consent obtained after checking eligibility.
Ethics oversight	University of Nottingham Ethics Committee ref 161-1711

Note that full information on the approval of the study protocol must also be provided in the manuscript.

Flow Cytometry

Plots

Confirm that:

☒ The axis labels state the marker and fluorochrome used (e.g. CD4-FITC).

☒ The axis scales are clearly visible. Include numbers along axes only for bottom left plot of group (a 'group' is an analysis of identical markers).

☒ All plots are contour plots with outliers or pseudocolor plots.

☒ A numerical value for number of cells or percentage (with statistics) is provided.

Methodology

Sample preparation	Macrophages were stained with 500nM 1 +/- 10microM ZM241385 for 2hrs at 37degrees then washed in the plate with cold PBS. Macrophages harvested by incubating on ice then acquired immediately by flow cytometry.
Instrument	Miltenyi MACSQuant
Software	Flowjo
Cell population abundance	At least 50,000 events collected per sample
Gating strategy	Debris was excluded using a FSC/SSC gate followed by doublet exclusion (FSC-A/FSC-H). All singlet macrophages were included in analysis of Cy5 fluorescence to indicate degree of binding of 1. Mean fluorescence intensity +/- SD was determined for each sample using Flowjo software.

☒ Tick this box to confirm that a figure exemplifying the gating strategy is provided in the Supplementary Information.
