# Supplementary material for: Ligand-directed covalent labelling of a GPCR with a fluorescent tag in live cells
Source: Commun Biol. 2020 Nov 27;3:722. doi: 10.1038/s42003-020-01451-w (PMC7695831; doi:10.1038/s42003-020-01451-w)
Supplement: Supplementary file 2 — Peer Review File [file 42003_2020_1451_MOESM2_ESM.pdf]

Reviewers' comments:

Reviewer #1 (Remarks to the Author):

This manuscript under consideration submitted by Leigh A. Stoddart et al is a stunning combination of well though chemistry and pharmacology that only a few unique labs or places can combine.

The team has designed a selective labeling tool of a fluorescently labeled ligand for a model GPCR which transfers its label to native receptors and even in cell lines naturally expressing the targeted receptor. Overall, I am impressed by the quality of work presented and have little to critizise.

Still, there are a few things that I would like the authors to address in a bit more detail.

1) Linker:
The authors state in their introduction that the generally Ser, Thr, or Lys can be a target for labeling and introduce their special Fluorine-labeled phenylester group. To me it is currently unclear why this label is selectively transferring it´s cargo to a Lys and is not attacked by Ser or Thr, in the vicinity of Lys153.
Can this selectivity in reactivity be explained a bit more?

2) Proposed position K153,
The docking in suppl figure 2 was performed using a truncated ligand without the fluorophore, which it transfers to the receptor. Since the fluorophore is actually bigger than the modeled ligand, it is unclear why this part should have no effect upon the relative orientation of the ligand within the binding pocket. In fact it might bring the ligand closer to K 150 and transfer the cargo to this amino acid.
Therefore, I would suggest to discuss this possibility or alternatively, create the K153A mutant to pin it down to this amino acid with experimental data.

Figure 2e
To be polite, I have seen much better pictures from this team when it comes to cell surface labeling…
Can the authors speculate a bit more about local clustering of the A2A receptor? If I recall correctly the same group showed a localized distribution for the A3 receptor using fluorescent ligands some time ago…

In my opinion, besides the above criticism, this study reflects an outstanding technical achievement and is very elegant work!

Reviewer #2 (Remarks to the Author):

Stoddart, Kindon et al., present a concise study where they report on fluorescent labelling of endogenous A2AR by using covalently bound fluorescent moiety of ZM241385 antagonist derivative. It is a nicely written piece on a methodology that could potentially be employed to other receptors

instead of e.g. tagging native receptors with CRISPR-Cas9. In fact, I do not see why it should not be accepted as it is. I think it will be of interest to the readership of Comms Bio. I only have two minor comments:

1. Fig 1a is misleading as it indicates that upon reaction of the electrophilic linker, the modified ZM241385 core of the compound 1 dissociates from the A2AR binding pocket. I believe that at saturating concentration both the fluorophore and the antagonist core are bound.
2. Figure 2g – I think median fluorescence intensity is preferable when logarithmic distribution (1 is missing on the x axis) of the channel intensity is used. Furthermore, it seems incorrect to use paired t-test in this case as the two treatments are independent of each other.

Reviewer #3 (Remarks to the Author):

The authors claim that they could rationally design a covalent and selective ligand probe to fluorescently label the A2A receptors in transiently transfected and endogenous systems. The synthesized compound (1) is a bivalent molecule with ZM241385 antagonist and Cy5 moiety connected by a phenyl ester-containing reactive linker, which is able to conjugate the fluorophore to K/S/Y amino acids through substitution reaction. The authors use TR-FRET to demonstrate the covalent reactivity of the probe with the A2A receptor, which is concentration-dependent and inhibited by co-incubation with an unlabeled antagonist. They used a CRE-mediated gene reporter system to show that cells treated with or without compound 1 are able to respond similarly to agonist CGS21680, suggesting that the binding site is liberated after covalent labeling. It would be interesting to see more characterization of the labeling kinetics for compound 1 in live cells.

The authors demonstrate the selectivity of compound 1 for A2A and not other adenosine receptor subtypes using live-cell confocal imaging. They claim that the selectivity can be attributed to the combination of selectivity of ZM241385 and the proximity of the nucleophilic residue K153 present only in the A2A receptor. It would be helpful to demonstrate by mutagenesis that K153 is indeed the attachment point for the fluorophore and responsible for the subtype selectivity. The authors should introduce a different amino acid in that position on A2A to see if the labeling is lessened. Moreover, they should test if by introducing lysine at a homologous site in the other receptor subtypes, they could rescue labeling.
It is not clear how efficient the reaction with residue types other than lysine, such as serine and tyrosine, would be under physiological conditions. What about other residue types, such as cysteine? Cysteines should be more reactive, but the thioester bonds would be more prone to hydrolysis. They could still contribute to label transfer reactions.

One of the main benefits they claim from their approach over other labeling methodologies is the ability to label receptors in endogenous systems. In my opinion, their endogenous labeling characterization a bit weak. The authors extend their labeling approach to endogenous A2A labeling in SK-BR-3 cells using live-cell confocal imaging and human monocyte-derived macrophages using flow cytometry. They indeed show differential fluorescent labeling in the presence and absence of compound 1 for both cell types. In the live-cell confocal labeling, the authors claim robust cell-surface labeling. What is the membrane permeability of compound 1? Presumably, to be able to use this approach to understand endogenous trafficking/signaling, it would be necessary to consider this permeability, as well as the effects of pH and media composition, may have on reactivity/efficiency/stability of labeling.

The ligand-directed approach is novel and has only been employed on a handful of GPCR targets within the last couple of years (https://doi.org/10.7554/eLife.49319.001). However, it does have several limitations over traditional labeling approaches, namely the need for a well-characterized binding site and known ligand scaffold for the receptor. ZM241385 is a highly potent A2A antagonist (Ki= 800 pM). That raises the question of whether this approach is robust enough to employ less potent ligands for directing the labeling.

Overall, the authors do a sufficient job supporting their claims with data. However, it would be good to expand the discussion of the limitations of the approach, especially since this presentation only weakly characterizes endogenous labeling, which seems to be the main benefit of the approach.

Dear Editor,

Many thanks for your eMail and attached referee reports. As requested, we have responded to each of the comments (as detail below) and have indicated in the attached revised manuscript (highlighted in yellow) the changes that we have made. As a consequence of some of the referee comments, we have split the two figures into four.

Reviewer #1.

This manuscript under consideration submitted by Leigh A. Stoddart et al is a stunning combination of well though chemistry and pharmacology that only a few unique labs or places can combine.

We thank the reviewer for these extremely positive comments. They are very much appreciated.

Specific points:

1. Linker: The authors state in their introduction that the generally Ser, Thr, or Lys can be a target for labeling and introduce their special Fluorine-labeled phenylester group. To me it is currently unclear why this label is selectively transferring it´s cargo to a Lys and is not attacked by Ser or Thr, in the vicinity of Lys153. Can this selectivity in reactivity be explained a bit more?

The ability of a serine or threonine residue to react with our fluorine-activated phenyl ester is indeed possible and as identified by the reviewer, we make appropriate commentary about this in the introduction to the manuscript. However, it is important to stress that within the surrounding vestibule of the A2a-orthosteric pocket there are surprisingly few nucleophilic side-chain residues. Notwithstanding this observation, the reviewer importantly highlights that additional discussion was needed in the manuscript and, in light of both these comments and those articulated in point 2 below, we undertook additional modelling studies with the entire ligand conjugate. Figure 1c (see below) in the main manuscript now includes a stereo-view of the top binding pose of compound **1** and has been labelled to highlight the approximate molecular distances between the electrophilic centre of the phenyl ester and the three nearest nucleophilic residues; Lys150, Lys153 and Ser156.

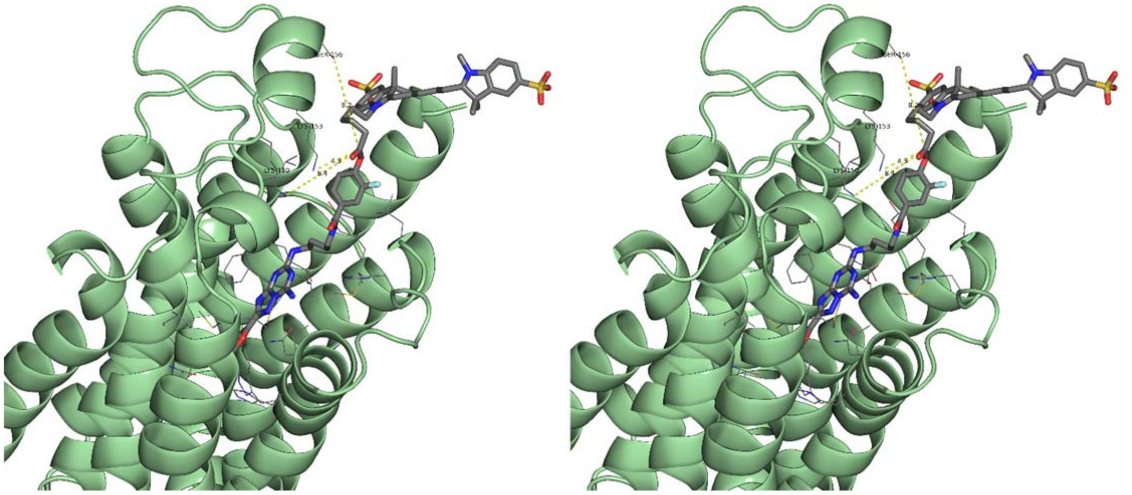

Figure 1c

2. Proposed position K153. The docking in suppl figure 2 was performed using a truncated ligand without the fluorophore, which it transfers to the receptor. Since the fluorophore is actually bigger than the modeled ligand, it is unclear why this part should have no effect upon the relative orientation of the ligand within the binding pocket. In fact it might bring the ligand closer to K 150 and transfer the cargo to this amino acid. Therefore, I would suggest to discuss this possibility or alternatively, create the K153A mutant to pin it down to this amino acid with experimental data.

Following on from the comments above, we have redesigned Figure 2 in the supplementary information (SI) document to provide further support to that of Figure 1. In Figure 2 of the SI we now show a range of top poses generated when docking compound **1** into the A2a receptor. As correctly identified by the reviewer, with the ZM element of our reagent bound in the orthosteric pocket the flexibility associated with the linker, when coupled with the charged Cy5 fluorophore, offers a number of possible conformations of the bound conjugate. However, the majority of these poses still reveal a close proximity between the electrophilic region of the linker and Lys153, with the fluorophore in a predominantly solvated state. With Ser156 consistently 8-10 Å from the carbonyl of the phenyl ester, we therefore maintain our reasoning that Lys153 remains the most realistic point of covalent transfer.

We have added the following text to line 60 of the text to discuss these points.
'The majority of the ligand-receptor poses revealed a close proximity (4-7 Å) of the ε-amino group of Lys153 and the electrophilic region of the linker and that the fluorophore was in a predominately solvated state. Within the surrounding vestibule of the orthosteric binding pocket of the A_{2A}AR there are surprisingly few nucleophilic side chain residues and the approximate molecular distances between the electrophilic centre of the phenyl ester and the two nearest nucleophilic residues; Lys150 and Ser156 were found to be consistently 8-10 Å from the carbonyl of the phenyl ester.'

3. Figure 2e. To be polite, I have seen much better pictures from this team when it comes to cell surface labeling…Can the authors speculate a bit more about local clustering of the A2A receptor? If I recall correctly the same group showed a localized distribution for the A3 receptor using fluorescent ligands some time ago.

We thank the reviewer for politely drawing our attention to the less obvious membrane labelling in Fig 2e, compared to those in either or previous publications, or perhaps within this publication in Figure 3. This is partly due to the difference in expression levels in the over-expressing HEK cell systems versus much lower levels of endogenous expression in SKBR3 cells, and partly due to their morphology. At these low levels, signal to noise ratio is reduced, particularly with the gain and offset on the microscope set to ensure the bright vesicular receptors are within the detector's dynamic range. Combined with the rather flat morphology of SKBR3 cells, we accept this does not make the normal "halo" of the plasma membrane as clear.

The reviewer is correct that we previously published a very plaque-like distribution of the A_3AR in human neutrophils (Corriden et al., EMBO Rep., 2013). The distribution seen here for the A_{2A}AR is not as extreme as for the A_3AR but is clearly clustered/vesicular. This may represent membrane clusters of receptors, but more likely shows labelled A_{2A}AR which has been constitutively trafficked by the vesicular machinery of the cells. We have slightly modified our description of the distribution in the manuscript. This aspect of the A_{2A}AR is not well studied, and we propose to use this ligand to further investigate this aspect of endogenous receptor organisation in future experiments.

To address this in the text, we removed 'clear cell surface' from line 143 and added in the following text to line 155 'When compared to the imaging observed in the HEK293 cells overexpressing SNAP-A_{2A}AR (Figure 3a), the labelling in the SK-BR-3 is more punctate. The SK-BR-3 cells have a flat morphology when compared HEK293 cells and as thus it is difficult to obtain a halo of plasma membrane labelling. In addition, there appears to be more intracellular clusters of receptors which may be due to labelled A_{2A}AR which has been constitutively trafficked by the vesicular machinery of the cells.'

Reviewer #2.

Stoddart, Kindon et al., present a concise study where they report on fluorescent labelling of endogenous A2AR by using covalently bound fluorescent moiety of ZM241385 antagonist derivative. It is a nicely written piece on a methodology that could potentially be employed to other receptors instead of e.g. tagging native receptors with CRISPR-Cas9. In fact, I do not see why it should not be accepted as it is. I think it will be of interest to the readership of Comms Bio.

Thank you for these comments.

Minor comments:

1. Fig 1a is misleading as it indicates that upon reaction of the electrophilic linker, the modified ZM241385 core of the compound 1 dissociates from the A2AR binding pocket. I believe that at saturating concentration both the fluorophore and the antagonist core are bound.

Whilst Figure 1 (a) states in point 3 that following covalent transfer of the fluorescent cargo, the antagonist is free to dissociate from the orthosteric pocket, this would need to be under diluting conditions (*e.g.* washing) in order to facilitate its removal. This is demonstrated and described in the manuscript through challenging the labelled receptor with the agonist CGS21680 (Figure 2d), resulting in an equally potent response when compared to untreated receptor. In order to ensure full clarity, however, we have changed the text in point 3 of figure 1a (see below) to include the requirement for washing out the orthosteric ligand and thank the reviewer for highlighting this.

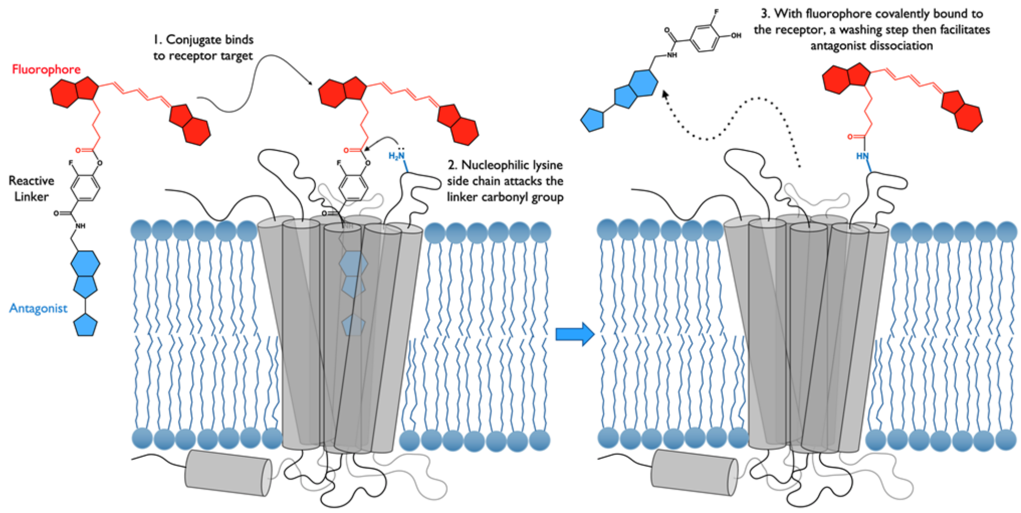

2. Figure 2g – I think median fluorescence intensity is preferable when logarithmic distribution (1 is missing on the x axis) of the channel intensity is used. Furthermore, it seems incorrect to use paired t-test in this case as the two treatments are independent of each other.

As requested, we have changed Figure 2g (now Figure 3b) to median intensity (See below) which has not changed the outcome for the analysis (p value 0.006 vs 0.007 using mean and median, respectively).This has been altered in the text (line 163).

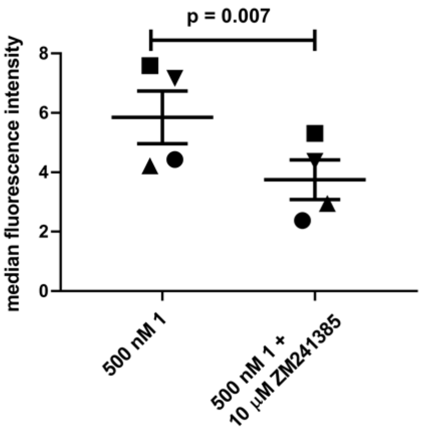

Figure 4b

We thank the reviewer for drawing our attention to the missing ‘1’ on the x-axis of Figure 2f. It had been plotted on a hyper-log scale (where 0-1 is shown as linear, and >1 as log) which is a proprietary function of the software used and is a common way to display FACS data. However, as it is not necessary here, we have converted Figure 2f (now Figure 4c) to a true log scale.

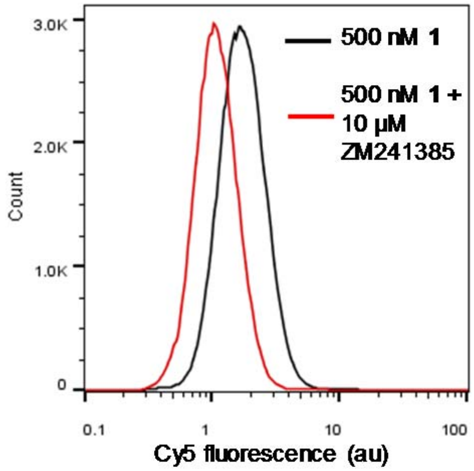

Figure 4c

We do, however, think that a paired t-test is the correct statistical analysis in this instance, since for each experiment a single set of macrophages were isolated from an individual donor, which were then split and then exposed to ligand either in the absence or presence of ZM241385. The data are therefore paired with respect to donor macrophage preparation. The paired t-test compares the change between treatments for a series of different donors.

Reviewer #3

1. It would be interesting to see more characterization of the labeling kinetics for compound 1 in live cells.

We have included data into Figure 2 of TR-FRET measurements taken at SNAP-A_{2A}AR in the presence two different concentrations of **1** at 1h, 3h and 5h (Figure 2b, see below). When using 250 nM **1**, maximal labelling was observed after 1h and no dissociation was observed after 2h of labelling in live cells, we believe that for this proof of principle study this indicates that full labelling has taken place after 2h.

From previous studies comparing binding kinetics at membrane and cells at the A_3AR (Bouzo-Lorenzo et al al, Purinergic Signalling, 2019) we found close agreement with the measured binding kinetics. When using **1** the measured binding kinetics will also incorporate ligand-receptor complexes where the ligand has bound, but not, labelled the receptor. We plan to explore the influence of compounds with more (or less) reactive cores in future work.

We have included the following text from line 76 to describe Figure 2b 'As these measurements were taken after 1h, to investigate if this was long enough to reach maximal labelling, TR-FRET measurements were also taken at 3h and 5h. Figure 2b demonstrates that there was no increase in the TR-FRET signal after 3h and 5h with 250nM **1,** whereas with lower concentrations a time-dependent increase was seen. This is expected, since the rate of association of the ligand is dependent on the concentration of ligand used, so for lower concentrations of ligand it will take longer to achieve an initial binding equilibrium and to then subsequently label the receptors. From this data, in further experiments the minimum labelling conditions were for 2h with 250nM **1.'**

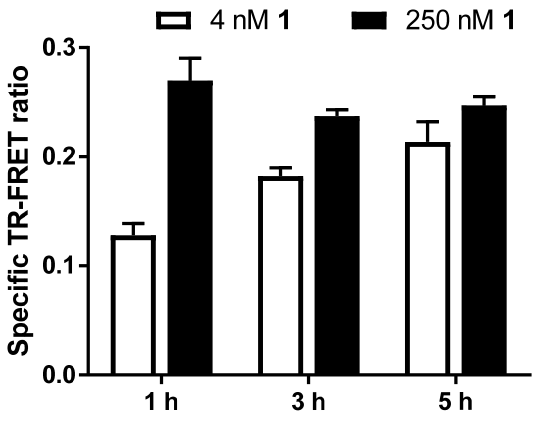

Figure 2b

2. The authors demonstrate the selectivity of compound 1 for A2A and not other adenosine receptor subtypes using live-cell confocal imaging. They claim that the selectivity can be attributed to the combination of selectivity of ZM241385 and the proximity of the nucleophilic residue K153 present only in the A2A receptor. It would be helpful to demonstrate by mutagenesis that K153 is indeed the attachment point for the fluorophore and responsible for the subtype selectivity. The authors should introduce a different amino acid in that position on A2A to see if the labeling is lessened. Moreover, they should test if

by introducing lysine at a homologous site in the other receptor subtypes, they could rescue labeling.

Whilst we agree with the reviewer that a definitive identification of the fluorophore attachment point would help pinpoint the ligand's binding pose and help with development of future ligands, we do not think this has a direct bearing on the main conclusions of our manuscript. For this reason, we believe that mutagenesis (to substitute K153 for other amino acids) is beyond the scope of the current manuscript, particularly given the fact that our labs have been in lock-down in the UK now for over four months (with no experiments allowed) and we have yet to return. ZM241385 is selective for A_{2A}AR, the low affinity for ZM241385 for the other adenosine receptors would require very high concentration of the ligand to be used, and the resulting binding kinetics would likely not allow long enough contact between the linker and nucleophile for the reaction to occur. It is the combination of subtype-selective ligand and proximity of a nucleophilic residue that is important here. We will of course explore in future studies the potential to label other adenosine receptor subtypes (with subtype selective probes) in a similar manner.

3. It is not clear how efficient the reaction with residue types other than lysine, such as serine and tyrosine, would be under physiological conditions. What about other residue types, such as cysteine? Cysteines should be more reactive, but the thioester bonds would be more prone to hydrolysis. They could still contribute to label transfer reactions.

We have dealt with this aspect in our response to reviewer 1 (points 1 & 2) to the effect that other nucleophilic side-chain residues could indeed react with the activated phenyl ester within our reagent. However, the distinct lack of such residues (including cysteines which are in fact all disulphide linked in the A2AR) within the receptor regions surrounding the orthosteric binding pocket, coupled with our molecular modelling predicting poses which position the reactive portion of the linker within 4-7 Å of the ε-amino group of Lys153, makes the latter the most liable to undergo fluorescent cargo transfer. In addition, we have added cysteine to the list of nucleophilic residues on line 36.

To address this comment and that of reviewer 1, we have added the following text to line 61 of the manuscript: 'The majority of the ligand-receptor poses revealed a close proximity (4-7 Å) of the ε-amino group of Lys153 and the electrophilic region of the linker and that the fluorophore was in a predominately solvated state. Within the surrounding vestibule of the orthosteric binding pocket of the A_{2A}AR there are surprisingly few nucleophilic side chain residues and the approximate molecular distances between the electrophilic centre of the phenyl ester and the two nearest nucleophilic residues; Lys150 and Ser156 were found to be consistently 8-10 Å from the carbonyl of the phenyl ester.'

One of the main benefits they claim from their approach over other labeling methodologies is the ability to label receptors in endogenous systems. In my opinion, their endogenous labeling characterization a bit weak. The authors extend their labeling approach to endogenous A2A labeling in SK-BR-3 cells using live-cell confocal imaging and human monocyte-derived macrophages using flow cytometry. They indeed show differential fluorescent labeling in the presence and absence of compound 1 for both cell types. In the

live-cell confocal labeling, the authors claim robust cell-surface labeling. What is the membrane permeability of compound 1? Presumably, to be able to use this approach to understand endogenous trafficking/signaling, it would be necessary to consider this permeability, as well as the effects of pH and media composition, may have on reactivity/efficiency/stability of labeling.

We have not measured the membrane permeability of compound **1**. However, the sulphonic acids present on the Sulfo-Cy5 would be ionised at physiological pH and therefore we would expect compound **1** to be poorly cell penetrant as previously described (https://pubs.acs.org/doi/10.1021/ja303931b). In addition, in our imaging experiments in HEK293 cells (for example in figure 2D) there is very little cytoplasmic signal indicating poor membrane permeability of compound **1** even after 2h of incubation. To understand endogenous receptor trafficking/signalling of cell surface receptors, the ideal compound would only label receptor at the cell surface to allow receptors that have been internalised to be selectively monitored.

We have also measured the stability of compound **1** in the physiological buffer used in all imaging experiments and found it had half-life of approximately 10h, which is in line with the reported half-lives for other compounds designed for ligand-directed chemistry (https://doi.org/10.1039/C5SC00190K).

The ligand-directed approach is novel and has only been employed on a handful of GPCR targets within the last couple of years (https://doi.org/10.7554/eLife.49319.001). However, it does have several limitations over traditional labeling approaches, namely the need for a well-characterized binding site and known ligand scaffold for the receptor. ZM241385 is a highly potent A2A antagonist (Ki= 800 pM). That raises the question of whether this approach is robust enough to employ less potent ligands for directing the labeling.

The ligand-directed approach compliments the very successful traditional labelling approaches such as the SNAP-tag and NanoLuc technologies. It does not require genetic manipulation of the cell and therefore, can be used to study endogenous receptors. We agree that this approach does require a known ligand and structural data about the target receptor greatly facilitates one’s ability to design a compound which delivers the fluorescent probe to a defined region of the receptor. The efficiency and stoichiometry of fluorescent probe transfer would be expected to depend upon both potency of the ligand and rate of ligand transfer. In the latter case, this will depend on the proximity of the reactive group on the ligand to the reactive amino acid. The closer the reactive group on the ligand is to the binding site entrance the less conformational freedom it will have; making fluorescent probe transfer to the receptor more efficient, so long as a suitable nucleophilic amino acid residue is appropriately located in the same vicinity. This may mean that, with very efficient probe transfer, a lower potency ligand could be used but may present the situation whereby the orthosteric binding site then becomes occluded by the fluorophore and limiting onward pharmacology. The ligand in the paper highlighted by Reviewer 3 which targets the μ opioid receptor has an affinity of ~60nM, which is nearly 100 times less than ZM241385, therefore it is likely that there is scope for this technique to be applied to ligands that have lower affinity. Extending this approach to other receptors and thoroughly investigation the full scope and limitations is the focus of ongoing work within our laboratories.

Overall, the authors do a sufficient job supporting their claims with data. However, it would be good to expand the discussion of the limitations of the approach, especially since this presentation only weakly characterizes endogenous labeling, which seems to be the main benefit of the approach.

Thank you for this comment. We have expanded the discussion of the limitations of the approach as requested.
The following text has been added starting at line 165: 'Ligand directed labelling of a GPCR compliments the very successful traditional labelling approaches such as the SNAP-tag[16] and NanoLuc[17] technologies. As an extension of studying GPCRs with fluorescent ligands, this means that each GPCR target requires a separate ligand-directed label to be developed[18]. With the explosion in the number of high-resolution structures solved[19], the rational design of ligand-directed labels for GPCRs should be achievable through the use of molecular modelling to reduce the size of the reactive groups and gain selectivity over closely related receptor subtypes through careful positioning of these groups close to subtype-specific nucleophilic residues. An often-observed pitfall with fluorescent ligands is high levels of non-specific binding, which can occlude the detection of specific binding in endogenously expressing systems[20]. As multiple wash steps can be performed in a ligand-directed labelling approach, this has the potential to reduce the levels of non-specific binding observed. Fluorescent ligands[21-23] and the recently described ligand-directed label for the μ opioid receptor[24] have been used to visualise the organisation and expression pattern of endogenously expressed receptors. Due to the proposed role of the $A_{2A}R$ in neurological conditions such as Parkinson's disease[25] and as a target for cancer immunotherapy[26], the approach described here has the potential to allow the function of the $A_{2A}R$ in normal and disease conditions to be studied.'

We trust that with these changes to the revised manuscript and the above responses to referees' comments that our paper is now acceptable for publication in Communications Biology.

With best wishes,

Steve Hill & Barrie Kellam

REVIEWERS' COMMENTS:

Reviewer #2 (Remarks to the Author):

I would like to thank the authors for providing the clarifications. Even though I agree to disagree with the statistical analysis, in my opinion, the manuscript can now be accepted.

Reviewer #3 (Remarks to the Author):

The changes to the revised manuscript sufficiently address my earlier comments. The paper will be a nice addition to Communications Biology.
